# Factors associated with potentially inappropriate medication use, medication underuse and overuse in older adults in the German National Cohort

**DOI:** 10.1111/joim.70097

**Published:** 2026-04-11

**Authors:** Miriam Degen, Ulrike Haug, Oliver H. F. Scholle, Yaw Awuku‐Larbi, Sigrid Carlsson, Nina Ebert, Volker Harth, Anja M. Hauri, Margit Heier, Anne Herrmann, Michael Leitzmann, Jakob Linseisen, Claudia Meinke‐Franze, Nadia Obi, Phil Pendt, Thomas Reinhold, Barbara Thorand, Hermann Brenner, Ben Schöttker

**Affiliations:** ^1^ Division of Clinical Epidemiology of Early Cancer Detection German Cancer Research Center (DKFZ) Heidelberg Germany; ^2^ Faculty of Medicine University of Heidelberg Heidelberg Germany; ^3^ Department of Clinical Epidemiology Leibniz Institute for Prevention Research and Epidemiology—BIPS Bremen Germany; ^4^ Faculty of Human and Health Sciences University of Bremen Bremen Germany; ^5^ Department of Epidemiology Helmholtz Centre for Infections Research Braunschweig Germany; ^6^ Department of Translational Medicine Division of Urological Cancers Lund University Lund Sweden; ^7^ Institute for Biometrics and Epidemiology German Diabetes Center Leibniz Center for Diabetes Research at the Heinrich Heine University Düsseldorf Düsseldorf Germany; ^8^ Institute for Occupational and Maritime Medicine (ZfAM) University Medical Center Hamburg‐Eppendorf Hamburg Germany; ^9^ Institute of Epidemiology Helmholtz Zentrum München German Research Center for Environmental Health (GmbH), Neuherberg Neuherberg Germany; ^10^ KORA Study Centre University Hospital of Augsburg Augsburg Germany; ^11^ Institute of Epidemiology and Preventive Medicine/Medical Sociology University of Regensburg Regensburg Germany; ^12^ Department of Internal Medicine III University Hospital Regensburg Germany; ^13^ Bavarian Cancer Research Center Regensburg Germany; ^14^ Institute of Epidemiology and Preventive Medicine University of Regensburg Regensburg Germany; ^15^ Institute of Epidemiology University of Augsburg Augsburg Germany; ^16^ Institute for Community Medicine University Medicine Greifswald Greifswald Germany; ^17^ Institute for Social Medicine Epidemiology and Health Economics Charité—Universitätsmedizin Berlin Berlin Germany; ^18^ Institute for Medical Information Processing Biometry and Epidemiology (IBE) Faculty of Medicine LMU Munich Pettenkofer School of Public Health Munich Germany; ^19^ German Center for Diabetes Research (DZD) Partner München‐Neuherberg Neuherberg Germany; ^20^ Cancer Prevention Graduate School German Cancer Research Center (DKFZ) Heidelberg Germany

**Keywords:** geriatrics, internal medicine, medication review, pharmacoepidemiology, quality of healthcare

## Abstract

**Background:**

Effectively identifying individuals exposed to drug underuse, overuse and potentially inappropriate medication (PIM) in older adults is essential for minimizing preventable drug‐related harms.

**Methods:**

We analysed data from 54,296 individuals aged 60–74 years from the German National Cohort (NAKO). We assessed the frequency of PIM, untreated conditions (medication and vaccination underuse) and medications without indication (overuse) utilizing the Fit fOR The Aged (FORTA) list. Factors associated with PIM, medication overuse and underuse were identified by multivariable logistic regression models.

**Results:**

The frequency of PIM, underuse and overuse of medications was 26.1%, 19.1% and 23.6%, respectively. In participants with available vaccination information, 90.6% and 62.7% did not have the recommended pneumococcal and annual influenza virus vaccinations, respectively. Parkinson's disease, arterial hypertension, epilepsy and depression were strongly associated with PIM use, with 16.2‐, 4.5‐, 4.3‐ and 2.1‐fold increased odds, respectively. Osteoporosis, atrial fibrillation, Parkinson's disease and chronic obstructive pulmonary disease were identified as particularly strongly associated with underuse, with 9.5‐, 6.1‐, 2.6‐ and 2.0‐fold increased odds, respectively. Depression and epilepsy were the most prominent factors associated with overuse (1.6‐ and 1.5‐fold increased odds).

**Conclusion:**

PIM and medication overuse, as well as drug and vaccine underuse, are very common among older German individuals. Comprehensive medication reviews are needed to improve medication quality. This work identifies people with specific characteristics who would benefit the most and could be prioritized for medication reviews.

## Graphical abstract

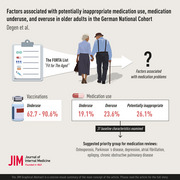

**Abbreviations**
BMI: body‐mass‐index
CI: confidence interval
COPD: chronic obstructive pulmonary disease
FORTA: Fit fOR The Aged
OR: odds ratio
PIM: potentially inappropriate medication
RCT: randomized controlled trial

## Background

Optimizing pharmacotherapy for older individuals is an ongoing challenge in our ageing society. In recent years, the focus has shifted away from merely reducing medication quantity to improving medication quality. This encompasses addressing three subdomains: overuse, underuse and drugs with an expected poor risk‐benefit ratio in older patients, so called potentially inappropriate medications (PIMs) [1, 2, 3].

Accumulating evidence linking suboptimal medication quality to poor health outcomes, such as functional impairments, hospitalizations and premature death [4, 5, 6, 7], indicates a necessity to improve overall medication quality in older adults. However, under‐ and overuse of medications have received little attention in prior research, and PIM use remains common, with a proportion ranging from 10.8% to 37.4% among older adults in the general German population [8, 9, 10, 11].

Among a broad variety of medication assessment tools [2, 3, 12, 13, 14, 15, 16, 17, 18, 19, 20], the Fit fOR The Aged (FORTA) list [20] addresses all three subdomains of suboptimal medication quality and is tailored to the medication availability and prescribing patterns in Germany. It has been coherently evaluated by a large number of multidisciplinary experts and is regularly updated, reflecting its consolidated validity and recent advances in geriatric pharmacotherapy, and has been validated in a randomized clinical trial [7].

The German National Cohort (NAKO) represents the largest German epidemiologic cohort study with recruitment in the whole country. The wide scope of available data, including self‐reported data, medical and functional assessments and blood‐based biomarkers [21], enables the investigation of potential factors associated with PIM use, medication underuse or overuse more comprehensively than in any previous study [8, 9, 10, 22, 23, 24, 25].

Therefore, we aimed to assess the frequency and factors associated with overuse, underuse and PIM use among older adults (60–74 years) who participated in the baseline assessment of the NAKO.

## Materials and methods

### Data source

We conducted a cross‐sectional study based on data from the baseline assessment visit of the NAKO, a large national, multi‐centre, population‐based, prospective cohort study that recruited approximately 205,000 participants aged 19 to 74 years in 18 assessment centres between 2014 and 2019. Detailed methods have been published elsewhere [21].

### Study population

Out of 204,666 participants with available baseline data, we included those aged 60–74 years, with available information on medication use, resulting in *n* = 54,296 participants eligible for our main analysis (Fig. S1). Thereof, *n* = 5575 participants fully completed the NAKO vaccination data module. The upper age cut‐off was given by the inclusion criteria of the NAKO, and the lower age cut‐off was chosen in a trade‐off between sample size and fit of the population. Although the age cut‐off of 65 years is more commonly used, 60 years has also been used in prior research on medication quality among older adults [6, 26, 27].

### Data collection

Sociodemographic information and lifestyle factors were collected through touchscreen questionnaires and interviews. Information about diseases was based on self‐reported data, where participants were asked if they had ever been diagnosed with a specific condition by a physician. The PHQ‐9 questionnaire was utilized to assess moderate to severe depressive symptoms (PHQ‐9 sum scores 15–19 and ≥20, respectively). Functional assessments (e.g., blood pressure measurement) were conducted during physical examinations.

Medication use was assessed by computer‐assisted, face‐to‐face interviews. Participants were asked to bring packages of the medications they had used in the past 7 days, and if they forgot, they were called by phone after the study centre visit. In the interview, additional information was collected for each medication (such as prescription drugs and regular use or as needed). Vaccination information was extracted from participants’ vaccination booklets and additional questionnaires.

### Assessment of medication quality: overuse, underuse and potentially inappropriate medication (PIM) use

We assessed medication quality considering the three subdomains, PIM use, underuse and overuse, as dichotomized exposures based on the 2024 version of the FORTA list, which classifies medications into four categories, encompassing A (indispensable), B (beneficial), C (questionable) and D (avoid) [20].

The three subdomains were defined as follows:

- Overuse: any FORTA medication without an appropriate indication.
- Underuse: any untreated FORTA indication. To determine the need for medication, we also incorporated clinical biomarkers, for example, HbA_1c_ levels in participants with diabetes, as detailed in Table S1.
- PIM: any medication classified as FORTA C or D used in participants with the according indication.

Out of 23 common geriatric indications listed in the FORTA list [20], we included 17 indications predominantly based on self‐reported lifetime history of diagnoses and occasionally complemented by additional criteria such as diagnostic tools. The detailed definitions and assessments in the NAKO are listed in Table S1. Acute bacterial infections, nausea/vomiting and constipation were not included because these are not chronic diseases requiring long‐term medication. Anaemia, incontinence, bipolar disorder and behavioural/psychological symptoms of dementia were not included since relevant information about these diagnoses was not available. Insomnia requiring medical treatment could not be distinguished from other types of insomnia and was therefore also excluded from our underuse assessment. Possible alternative medications and indications not included in the FORTA list were also considered to mitigate the risk of misclassification of over‐ and underuse. The full list of potential medications to be used for these 17 indications and the assessed vaccinations is provided in Table S2.

We only included pneumococcal and annual influenza vaccines in our vaccination assessment because herpes zoster, COVID‐19 and respiratory syncytial virus (RSV) vaccines were not part of the national recommendations for vaccination among older adults in the entire recruitment period (2014–2019). The recommendations for herpes zoster, COVID‐19 and RSV vaccinations were announced later in 12/2018, 12/2020 and 04/2025, respectively [28, 29, 30]. Due to the small proportion of participants with complete available information on vaccinations (10.3%), we reported underuse of vaccines separately from underuse of other medications. We assessed the representativeness of this subpopulation with vaccination data by comparing the baseline characteristics of the included subsample with the baseline characteristics of those not included, and no major differences were observed (Table S3).

### Statistical analyses

We used multivariable logistic regression models to identify factors associated with PIM use, underuse, and overuse of medications as dependent variables. Results were presented as odds ratios (ORs) with 95% confidence intervals. Overall, 32 factors, including sociodemographic/‐economic factors, lifestyle factors, study centre and diseases, were selected based on subject matter knowledge. Multicollinearity and model stability were tested with the variation inflation index. After exclusion of self‐perceived general health status, no variable had a variance inflation factor ≥8. Therefore, the full model was adjusted for 31 independent variables, which are shown in Table 1. Additionally, models only adjusted for age, sex and study centre were computed.

**Table 1:** Distribution of potential factors associated with (PIM) use, medication underuse and overuse in the analysed study population of the NAKO (N = 54,296).

Characteristics	*N* (%)^a^	Proportion (%)^a^ of PIM users	Proportion (%)^a^ of individuals with underuse	Proportion (%)^a^ of individuals with overuse
**Sociodemographic/‐economic factors**				
Sex	—			
Female	27,189 (50.1)	27.4	21.0	26.1
Male	27,107 (49.9)	24.8	17.2	22.0
Age group, years	—			
60–64	25,853 (47.6)	24.0	19.3	21.7
65–69	24,121 (44.4)	27.6	18.8	24.8
≥70	4322 (8.0)	30.6	19.5	28.1
Study centre	‐			
Augsburg	5513 (10.2)	25.6	20.6	23.5
Regensburg	2678 (4.9)	28.6	18.5	27.6
Mannheim	2558 (4.7)	24.8	18.7	25.8
Freiburg	2654 (4.9)	20.2	21.9	21.3
Saarbrücken	2723 (5.0)	25.5	20.3	26.4
Essen	2670 (4.9)	27.5	21.8	25.7
Münster	2557 (4.7)	21.3	19.3	24.2
Düsseldorf	2364 (4.4)	24.5	19.4	27.4
Halle	2807 (5.2)	34.3	17.4	24.5
Leipzig	2840 (5.2)	32.6	20.1	20.9
Berlin North	2684 (4.9)	24.5	18.7	20.4
Central Berlin	2772 (5.1)	23.8	19.7	22.6
Berlin South	2718 (5.0)	21.9	19.0	23.4
Hannover	2866 (5.3)	24.0	18.6	22.7
Hamburg	2595 (4.8)	20.5	19.0	23.2
Bremen	2753 (5.1)	21.8	18.6	19.0
Kiel	2511 (4.6)	25.4	17.8	23.7
Neubrandenburg	3401 (6.3)	33.3	16.6	23.2
Neustelitz	809 (1.5)	31.9	18.9	21.3
Waren (Müritz)	1125 (2.1)	33.0	13.0	23.3
Demmin	698 (1.3)	36.5	17.2	25.8
Income^b^	—			
Low	30,479 (56.1)	30.4	20.4	24.7
Middle	16,866 (31.1)	22.0	17.8	22.1
High	6951 (12.8)	17.3	16.6	22.1
Years of education	—			
<13	2251 (4.1)	35.1	23.4	27.6
≥13	52,045 (95.9)	25.7	18.9	23.4
Social network index^c^	—			
I + II	29,454 (54.2)	27.6	20.0	24.2
III + IV	24,842 (45.8)	24.3	18.0	22.7
**Lifestyle factors**				
BMI (kg/m^2^)	—			
<20	1177 (2.2)	16.4	21.5	22.9
20–24.9	14,569 (26.8)	17.5	19.5	22.0
25–29.9	22,728 (41.9)	24.2	18.7	22.5
≥30	15,822 (29.1)	37.6	19.2	26.6
Physical activity level^d^	—			
Insufficient	6896 (12.7)	32.1	19.0	26.1
Sufficient	47,400 (87.3)	25.2	19.1	23.2
Alcohol consumption^e^	—			
Abstainer	5126 (9.4)	35.8	22.2	29.8
Low	43,113 (79.4)	25.1	18.7	22.7
Moderate to high	6057 (11.2)	25.3	19.2	24.3
Smoking status	—			
Never smoker	23,614 (43.5)	25.3	18.5	22.2
Former smoker, years since quit				
>20 years	12,799 (23.6)	25.3	18.8	22.3
≤20 years	8810 (16.2)	27.9	20.3	26.9
Current smoker	9073 (16.7)	27.7	19.9	25.7
**Comorbidities**				
Diabetes	6684 (12.3)	47.4	20.9	30.6
Arterial hypertension	26,961 (49.7)	40.8	21.4	25.1
Atrial fibrillation/Arrhythmia	8235 (15.2)	38.5	47.0	22.7
Stroke	1879 (3.5)	45.1	26.4	29.9
Myocardial infarction	2234 (4.1)	36.6	17.8	25.7
Heart failure	2976 (5.5)	43.1	31.2	30.3
Acute coronary syndromes	4391 (8.1)	34.4	20.5	24.8
Asthma	3944 (7.3)	31.8	27.7	25.3
COPD	4069 (7.5)	38.7	35.0	27.8
Osteoporosis	3955 (7.3)	36.7	61.1	24.9
Thyroid disease/Hypothyroidism^f^	14,951 (27.5)	30.5	21.8	26.7
History of fractures^g^	9703 (17.9)	27.6	24.6	23.7
Pain^h^	36,887 (67.9)	29.8	21.7	23.9
Parkinson's disease	197 (0.4)	80.2	38.6	31.0
Cognitive impairment/Dementia^i^	2234 (4.1)	41.0	30.2	24.1
History of depression^j^	8531 (15.7)	42.1	26.2	32.5
Epilepsy	396 (0.7)	60.1	22.0	34.1
Insomnia	13,836 (25.5)	29.1	22.3	23.5
Gastrointestinal symptoms^k^	16,540 (30.5)	34.1	26.4	24.6
History of cancer	7612 (14.0)	29.3	20.9	26.5
Renal impairment^l^	2728 (5.0)	41.5	18.3	33.4

Abbreviations: BMI, body mass index; COPD, chronic obstructive pulmonary disease; PIM, potentially inappropriate medication.

^a^ Calculated from first imputed dataset.

^b^ Average total household income before tax. Categories: low: <€2900; middle: €2900–<€5000; high: ≥€5000.

^c^ Categories: I (isolated) to IV (least isolated).

^d^ Adherence to WHO recommendations following global physical activity questionnaire (GPAQ). Categories: sufficient: ≥600 metabolic equivalent of task (MET)–min/week; insufficient: <600 MET–min/week.

^e^ Alcohol consumption, g ethanol per day. Categories: abstainer: 0 g/day; low: women 0–19.99 g/day or men 0–39.99 g/day; moderate–high: women ≥20 g/day or men ≥40 g/day.

^f^ Lifetime history of thyroid disease, self‐reported. Underuse for the indication hypothyroidism was only considered in individuals with TSH > 8.0 mU/L (cut‐off for treatment of older patients suggested by FORTA list).

^g^ Fractures since aged 50 years.

^h^ Pain‐related diagnoses included certain or likely migraine or tension headache in the last 12 months (following the third edition of The International Classification of Headache Disorders), and self‐reported information on arthritis, osteoarthritis, and gout.

^i^ Based on the interview question ‘Have you ever contacted a physician due to memory difficulties or are you planning to speak to a physician about it?’.

^j^ Lifetime history of depression, self‐reported.

^k^ Combination of self‐reported information on reflux, peptic ulcer ('Have you ever been diagnosed with this condition by a physician?') and regular intake of NSAIDs (ATC M01A). Underuse for this indication was only considered if the participant was diagnosed with reflux or peptic ulcer in the year of their baseline assessment and in participants regularly taking NSAIDs.

^l^ Estimated glomerular filtration rate <60 mL/min/1.73 m^2^, calculated based on CKD‐EPI equation.

We utilized multiple imputations with the fully conditional specification method [31] and created five imputed datasets to handle missing covariates. Indications for medication use were not imputed. Instead, we assumed the absence of the indication if this information was missing. No covariate had more than 30% of missing values. The proportions of missing values for imputed covariates (Table S4) and medical indications (Table S5) are listed in the appendix. Details about the imputation procedure are outlined in Text S1.

We used SAS statistical software (version 9.4, SAS Institute, Inc.) for the statistical analyses. All statistical tests were two‐tailed, with a significance level set at *α* = 0.05 and *p*‐values adjusted for multiple testing using the false discovery rate [32] per model and outcome.

## Results

### Characteristics of the study population

The baseline characteristics of the study population (*n* = 54,296) are presented in Table 1. The median age was 65 years (interquartile range: 62–67), and half of the population was female (50.1%). The proportion of PIM users, individuals with underuse, and individuals with overuse in the total study population was 26.1%, 19.1% and 23.6%, respectively. Table 1 also shows descriptively how these proportions vary according to baseline characteristics.

### Frequency of and factors associated with PIM use

For the participants (*N* = 14,182 (26.1%)) using at least one PIM, the ranking of the proportions according to indication is presented in Table 2. The largest proportions of individuals taking a PIM to treat the specific indication were observed among patients with Parkinson's disease (59.9%), epilepsy (39.9%), arterial hypertension (27.1%), atrial fibrillation (23.4%) and a history of depression (22.4%).

**Table 2:** Ranking of proportion of participants with PIM use per indication in NAKO participants aged 60–74 years (N = 54,296).

Indication	*N*_indication_ (%)	*N* (%) with PIM use for specific indication
Parkinson's disease	197 (0.4)	118 (59.9)
Epilepsy	396 (0.7)	158 (39.9)
Arterial hypertension	26,961 (49.7)	7295 (27.1)
Atrial fibrillation/Arrhythmia	8235 (15.2)	1931 (23.4)
History of depression^a^	8745 (16.1)^b^	1963 (22.4)
Heart failure	2976 (5.5)	398 (13.4)
Cognitive impairment/Dementia^c^	2234 (4.1)	250 (11.2)
Pain^d^	36,887 (67.9)	3838 (10.4)
Myocardial infarction	2234 (4.1)	203 (9.1)
Diabetes	6684 (12.3)	571 (8.5)
Osteoporosis	3955 (7.3)	268 (6.8)
COPD	4069 (7.5)	257 (6.3)
Acute coronary syndromes	4391 (8.1)	273 (6.2)
Insomnia	13,836 (25.5)	814 (5.9)
Gastrointestinal symptoms^e^	16,540 (30.5)	101 (0.6)
Stroke	1879 (3.5)	11 (0.6)
Thyroid disease/Hypothyroidism^f^	14,951 (27.5)	na^f^

*Note*: PIM exposure was defined according to the ‘Fit fOR The Aged’ (FORTA) list 2024.

Abbreviations: COPD, chronic obstructive pulmonary disease; na, not available; PIM, potentially inappropriate medication.

^a^ Lifetime history of depression, self‐reported.

^b^ Including 8531 participants with self‐reported history of depression and 214 additional participants with moderate to severe depressive symptoms according to PHQ‐9.

^c^ Based on the interview question ‘Have you ever contacted a physician due to memory difficulties or are you planning to speak to a physician about it?’.

^d^ Pain‐related diagnoses included certain or likely migraine or tension headache in the last 12 months (following the third edition of The International Classification of Headache Disorders), and self‐reported information on arthritis, osteoarthritis and gout.

^e^ Combination of self‐reported information on reflux, peptic ulcer (Have you ever been diagnosed with this condition by a physician?) and regular intake of NSAIDs (ATC M01A).

^f^ Lifetime history of thyroid disease, self‐reported. No PIM defined for this indication.

The results of the age, sex and study centre–adjusted logistic regression model of potential factors associated with PIM use are provided in Table S6. Table 3 lists the results of the full model. Among the sociodemographic/‐economic factors, we observed 12% lower odds for PIM use in males compared to females. In participants aged 65–69 and ≥70 years, 10% and 17% higher odds for PIM use were observed, respectively, compared to those aged 60–64 years. Furthermore, participants with middle and high income had 11% and 19% lower odds for using PIM relative to participants with low income. We observed 14% lower odds for PIM use among participants with ≥13 years compared to <13 years of education. We also observed differences in the detected PIM rates in the 21 study centres. Compared to Augsburg, which was the largest study centre and recruited 10.2% of the study participants, 8 study centres had statistically significantly higher or lower odds for PIM use, with a range from 13% lower odds in Hamburg to 49% increased odds in Neubrandenburg.

**Table 3:** Results of the full logistic regression model of potential factors associated with PIM use, medication underuse and overuse in the NAKO study (N = 54,296).

	PIM, full model			Underuse, full model			Overuse, full model		
Variables	OR (95% CI)	*p* ^a^	FDR^b^	OR (95% CI)	*p* ^a^	FDR^b^	OR (95% CI)	p^a^	FDR^b^
**Sociodemographic/‐economic factors**									
Sex									
Female	Ref.			Ref.			Ref.		
Male	**0.88 (0.84; 0.92)**	**<0.001**	**<0.001**	**1.08 (1.02; 1.14)**	**0.010**	**0.026**	**0.75 (0.71; 0.78)**	**<0.001**	**<0.001**
Age group, years									
60–64	Ref.			Ref.			Ref.		
65–69	**1.10 (1.06; 1.16)**	**<0.001**	**<0.001**	**0.84 (0.80; 0.89)**	**<0.001**	**<0.001**	**1.21 (1.16; 1.27)**	**<0.001**	**<0.001**
≥70	**1.17 (1.08; 1.27)**	**<0.001**	**<0.001**	**0.75 (0.68; 0.83)**	**<0.001**	**<0.001**	**1.46 (1.35; 1.57)**	**<0.001**	**<0.001**
Study centre									
Augsburg	Ref.			Ref.			Ref.		
Regensburg	**1.15 (1.02; 1.29)**	**0.020**	**0.031**	**0.85 (0.75; 0.97)**	**0.019**	**0.044**	**1.21 (1.09; 1.35)**	**0.001**	**0.001**
Mannheim	0.96 (0.85; 1.09)	0.534	0.585	**0.85 (0.74; 0.97)**	**0.018**	**0.043**	1.10 (0.99; 1.23)	0.080	0.138
Freiburg	**0.85 (0.75; 0.96)**	**0.009**	**0.015**	1.13 (0.99; 1.28)	0.067	0.112	0.91 (0.81; 1.02)	0.090	0.147
Saarbrücken	0.99 (0.88; 1.12)	0.884	0.899	0.88 (0.78; 1.00)	0.056	0.097	**1.20 (1.08; 1.34)**	**0.001**	**0.002**
Essen	1.08 (0.96; 1.22)	0.173	0.225	0.99 (0.87; 1.13)	0.876	0.892	1.12 (1.00; 1.24)	0.049	0.094
Münster	0.97 (0.86; 1.10)	0.665	0.702	0.94 (0.83; 1.08)	0.386	0.468	1.10 (0.99; 1.23)	0.086	0.145
Düsseldorf	0.96 (0.85; 1.09)	0.515	0.575	0.87 (0.76; 1.00)	0.048	0.086	**1.20 (1.07; 1.34)**	**0.001**	**0.003**
Halle	**1.42 (1.27; 1.59)**	**<0.001**	**<0.001**	**0.67 (0.58; 0.76)**	**<0.001**	**<0.001**	1.04 (0.94; 1.16)	0.445	0.539
Leipzig	**1.33 (1.19; 1.48)**	**<0.001**	**<0.001**	0.86 (0.76; 0.98)	0.023	0.051	**0.87 (0.78; 0.97)**	**0.016**	**0.036**
Berlin North	0.93 (0.82; 1.05)	0.221	0.274	**0.80 (0.70; 0.91)**	**0.001**	**0.003**	**0.87 (0.78; 0.98)**	**0.019**	**0.043**
Central Berlin	0.89 (0.79; 1.00)	0.050	0.073	0.87 (0.76; 0.98)	0.028	0.057	0.93 (0.84; 1.04)	0.229	0.326
Berlin South	0.88 (0.78; 1.00)	0.045	0.067	0.86 (0.76; 0.98)	0.025	0.054	0.95 (0.85; 1.07)	0.411	0.521
Hannover	1.01 (0.89; 1.13)	0.928	0.928	**0.85 (0.75; 0.97)**	**0.016**	**0.040**	0.97 (0.86; 1.08)	0.524	0.621
Hamburg	**0.87 (0.77; 0.98)**	**0.026**	**0.040**	0.93 (0.82; 1.06)	0.304	0.400	0.97 (0.87; 1.09)	0.621	0.708
Bremen	0.97 (0.86; 1.09)	0.604	0.650	0.95 (0.84; 1.08)	0.467	0.544	**0.78 (0.69; 0.87)**	**<0.001**	**<0.001**
Kiel	1.07 (0.95; 1.21)	0.292	0.354	**0.77 (0.67; 0.88)**	**<0.001**	**0.001**	1.03 (0.92; 1.15)	0.655	0.732
Neubrandenburg	**1.49 (1.34; 1.66)**	**<0.001**	**<0.001**	**0.74 (0.65; 0.84)**	**<0.001**	**<0.001**	1.02 (0.92; 1.13)	0.758	0.822
Neustelitz	1.13 (0.95; 1.36)	0.167	0.222	0.81 (0.65; 1.00)	0.047	0.086	0.90 (0.75; 1.08)	0.262	0.355
Waren (Müritz)	**1.28 (1.10; 1.50)**	**0.002**	**0.003**	**0.51 (0.41; 0.62)**	**<0.001**	**<0.001**	1.00 (0.86; 1.17)	0.998	0.998
Demmin	**1.47 (1.22; 1.77)**	**<0.001**	**<0.001**	**0.61 (0.48; 0.77)**	**<0.001**	**<0.001**	1.12 (0.93; 1.35)	0.242	0.337
Income^c^									
Low	Ref.			Ref.			Ref.		
Middle	**0.89 (0.84; 0.94)**	**<0.001**	**<0.001**	0.95 (0.90; 1.01)	0.102	0.161	1.00 (0.95; 1.05)	0.837	0.867
High	**0.81 (0.74; 0.87)**	**<0.001**	**<0.001**	0.97 (0.89; 1.05)	0.426	0.506	1.07 (1.00; 1.15)	0.067	0.123
Years of education									
<13	Ref.			Ref.			Ref.		
≥13	**0.86 (0.77; 0.95)**	**0.004**	**0.007**	0.92 (0.82; 1.04)	0.188	0.274	1.00 (0.91; 1.10)	0.994	0.998
Social network index^d^									
I + II	Ref.			Ref.			Ref.		
III + IV	0.98 (0.93; 1.03)	0.437	0.509	0.99 (0.92; 1.06)	0.688	0.740	0.98 (0.92; 1.04)	0.534	0.621
**Lifestyle factors**									
BMI, kg/m^2^									
<20	1.09 (0.91; 1.30)	0.336	0.399	1.02 (0.86; 1.21)	0.843	0.874	1.02 (0.88; 1.18)	0.784	0.827
20–24.9	Ref.			Ref.			Ref.		
25–29.9	**1.12 (1.05; 1.19)**	**<0.001**	**<0.001**	0.96 (0.90; 1.02)	0.196	0.279	1.02 (0.97; 1.08)	0.373	0.494
≥30	**1.38 (1.30; 1.47)**	**<0.001**	**<0.001**	**0.82 (0.76; 0.88)**	**<0.001**	**<0.001**	**1.14 (1.07; 1.21)**	**<0.001**	**<0.001**
Physical activity level^e^									
Insufficient	Ref.			Ref.			Ref.		
Sufficient	**0.84 (0.79; 0.90)**	**<0.001**	**<0.001**	1.04 (0.96; 1.13)	0.341	0.423	**0.89 (0.84; 0.95)**	**<0.001**	**0.001**
Alcohol consumption^f^									
Abstainer	**1.29 (1.20; 1.40)**	**<0.001**	**<0.001**	0.98 (0.90; 1.06)	0.591	0.647	**1.28 (1.20; 1.38)**	**<0.001**	**<0.001**
Low	Ref.			Ref.			Ref.		
Moderate–high	1.03 (0.96; 1.10)	0.475	0.542	1.04 (0.96; 1.13)	0.272	0.369	1.07 (1.00; 1.14)	0.044	0.086
Smoking									
Never	Ref.			Ref.			Ref.		
Former smoker, years since quit									
>20 years	1.04 (0.98; 1.10)	0.201	0.254	1.02 (0.96; 1.08)	0.584	0.647	1.06 (1.01; 1.12)	0.024	0.051
≤20 years	1.05 (0.98; 1.12)	0.150	0.209	1.05 (0.98; 1.13)	0.187	0.274	**1.31 (1.23; 1.39)**	**<0.001**	**<0.001**
Current smoker	**1.16 (1.08; 1.25)**	**<0.001**	**<0.001**	1.03 (0.95; 1.10)	0.508	0.580	**1.24 (1.17; 1.32)**	**<0.001**	**<0.001**
**Comorbidities**									
Diabetes	**1.79 (1.69; 1.90)**	**<0.001**	**<0.001**	1.04 (0.96; 1.12)	0.309	0.400	**1.35 (1.27; 1.44)**	**<0.001**	**<0.001**
Arterial hypertension	**4.50 (4.28; 4.72)**	**<0.001**	**<0.001**	**1.29 (1.22; 1.36)**	**<0.001**	**<0.001**	**1.08 (1.03; 1.13)**	**0.001**	**0.002**
Atrial fibrillation/Arrhythmia	**1.52 (1.44; 1.61)**	**<0.001**	**<0.001**	**6.13 (5.79; 6.49)**	**<0.001**	**<0.001**	**0.82 (0.77; 0.87)**	**<0.001**	**<0.001**
Stroke	**1.40 (1.26; 1.56)**	**<0.001**	**<0.001**	1.14 (1.01; 1.28)	0.037	0.073	**1.23 (1.11; 1.37)**	**<0.001**	**<0.001**
Myocardial infarction	**1.54 (1.33; 1.77)**	**<0.001**	**<0.001**	0.88 (0.75; 1.05)	0.151	0.232	1.10 (0.96; 1.27)	0.183	0.268
Heart failure	**1.19 (1.09; 1.31)**	**<0.001**	**<0.001**	1.02 (0.92; 1.12)	0.759	0.801	**1.36 (1.25; 1.49)**	**<0.001**	**<0.001**
Acute coronary syndromes	**0.61 (0.55; 0.68)**	**<0.001**	**<0.001**	**0.67 (0.59; 0.75)**	**<0.001**	**<0.001**	**0.86 (0.78; 0.96)**	**0.007**	**0.017**
Asthma	0.93 (0.85; 1.01)	0.072	0.103	1.08 (0.99; 1.18)	0.085	0.139	0.99 (0.91; 1.07)	0.764	0.822
COPD	**1.37 (1.26; 1.48)**	**<0.001**	**<0.001**	**2.02 (1.86; 2.20)**	**<0.001**	**<0.001**	1.06 (0.98; 1.15)	0.117	0.185
Osteoporosis	**1.47 (1.36; 1.60)**	**<0.001**	**<0.001**	**9.51 (8.80; 10.29)**	**<0.001**	**<0.001**	0.93 (0.86; 1.01)	0.076	0.135
Thyroid disease/Hypothyroidism^g^	1.04 (0.99; 1.09)	0.160	0.218	0.94 (0.89; 1.00)	0.048	0.086	**1.10 (1.05; 1.15)**	**<0.001**	**<0.001**
History of fractures^h^	1.00 (0.94; 1.08)	0.884	0.899	1.04 (0.96; 1.11)	0.332	0.421	0.97 (0.91; 1.04)	0.381	0.494
Pain^i^	**1.40 (1.33; 1.47)**	**<0.001**	**<0.001**	**1.39 (1.31; 1.47)**	**<0.001**	**<0.001**	**0.91 (0.87; 0.95)**	**<0.001**	**<0.001**
Parkinson's disease	**16.23 (11.01; 23.91)**	**<0.001**	**<0.001**	**2.60 (1.85; 3.64)**	**<0.001**	**<0.001**	1.26 (0.92; 1.72)	0.151	0.232
Cognitive impairment/Dementia^j^	**1.76 (1.59; 1.94)**	**<0.001**	**<0.001**	**1.50 (1.34; 1.67)**	**<0.001**	**<0.001**	0.89 (0.81; 0.99)	0.031	0.063
History of depression^k^	**2.11 (1.99; 2.23)**	**<0.001**	**<0.001**	**1.12 (1.05; 1.19)**	**0.001**	**0.003**	**1.62 (1.54; 1.71)**	**<0.001**	**<0.001**
Epilepsy	**4.29 (3.40; 5.43)**	**<0.001**	**<0.001**	0.83 (0.63; 1.10)	0.202	0.281	**1.50 (1.21; 1.86)**	**<0.001**	**0.001**
Insomnia	**1.16 (1.10; 1.22)**	**<0.001**	**<0.001**	**1.14 (1.08; 1.21)**	**<0.001**	**<0.001**	0.97 (0.92; 1.02)	0.183	0.268
Gastrointestinal symptoms^l^	**1.37 (1.31; 1.43)**	**<0.001**	**<0.001**	**1.57 (1.49; 1.65)**	**<0.001**	**<0.001**	0.98 (0.94; 1.03)	0.428	0.530
History of cancer	**1.10 (1.04; 1.17)**	**0.002**	**0.003**	1.00 (0.94; 1.07)	0.939	0.939	**1.14 (1.07; 1.20)**	**<0.001**	**<0.001**
Renal impairment^m^	**1.57 (1.43; 1.72)**	**<0.001**	**<0.001**	**0.75 (0.67; 0.84)**	**<0.001**	**<0.001**	**1.43 (1.31; 1.56)**	**<0.001**	**<0.001**

*Note*: Values in bold are statistically significant with FDR (*p* < 0.05). PIM exposure was defined according to the ‘Fit fOR The Aged’ (FORTA) list 2024. All models were adjusted for sex, age group (60–64, 65–69, ≥70 years), study centre, income (1700–<€2900, €2900–<€5000, ≥€5000), years of education (<13, ≥13), social network indexd (I + II, III + IV), BMI group (<20, 20–24.9, 25–29.9, ≥30 kg/m^2^), physical activity level (insufficient, sufficient), alcohol consumption (abstainer, low, moderate‐to‐high alcohol consumption), smoking (never, former smoker with >20 years since quit, ≤20 years since quit, current smoker) and comorbidities (diabetes, arterial hypertension, atrial fibrillation/arrhythmia, stroke, myocardial infarction, heart failure, acute coronary syndromes, asthma, COPD, osteoporosis, thyroid disease/hypothyroidism, history of fractures, pain, Parkinson's disease, cognitive impairment/dementia, history of depression, epilepsy, insomnia, gastrointestinal symptoms, history of cancer and renal impairment).

Abbreviations: BMI, body mass index; CI, confidence interval; COPD, chronic obstructive pulmonary disease; FDR, false discovery rate; OR, odds ratio; PIM, potentially inappropriate medication; Ref., reference.

^a^ Unadjusted for multiple testing.

^b^ *p* values adjusted for multiple testing using the false discovery rate (Benjamini–Hochberg method).

^c^ Average total household income before tax. Categories: low: 1700–<€2900; middle: €2900‐<€5000; high: ≥€5000.

^d^ Categories: I (isolated) to IV (least isolated).

^e^ Adherence to WHO recommendations following global physical activity questionnaire (GPAQ). Categories: sufficient: ≥600 metabolic equivalent of task (MET)–min/week; insufficient: <600 MET–min/week.

^f^ Alcohol consumption, g ethanol per day. Categories: abstainer: 0 g/day; low: women 0–19.99 g/day or men 0–39.99 g/day; moderate–high: women ≥20 g/day or men ≥40 g/day.

^g^ Lifetime history of thyroid disease, self‐reported. Underuse was only considered in individuals with TSH > 8.0 mU/L (cut‐off for treatment of older patients suggested by FORTA list).

^h^ Fractures since aged 50.

^i^ Pain‐related diagnoses included certain or likely migraine or tension headache in the last 12 months (following the third edition of The International Classification of Headache Disorders), and self‐reported information on arthritis, osteoarthritis and gout.

^j^ Based on the interview question ‘Have you ever contacted a physician due to memory difficulties or are you planning to speak to a physician about it?’.

^k^ Self‐reported lifetime history.

^l^ Combination of self‐reported information on reflux, peptic ulcer ('Have you ever been diagnosed with this condition by a physician?') and regular intake of NSAIDs (ATC M01A). Underuse was only considered if the participant was diagnosed with reflux or peptic ulcer in the year of their baseline assessment and in participants regularly taking NSAIDs.

^m^ Estimated glomerular filtration rate <60 mL/min/1.73 m^2^, calculated based on CKD‐EPI equation.

Obesity (defined as body‐mass‐index [BMI] ≥ 30; 38% increased odds compared to normal BMI) and alcohol abstinence (29% increased odds compared to low alcohol consumption) were the lifestyle risk factors with the strongest association with PIM use. We further identified overweight (BMI 25–29.9) and current smoking as additional associated factors, with 12% and 16% increased odds for PIM use. Conversely, participants with sufficient physical activity had 16% lower odds for PIM use compared to participants with insufficient physical activity.

Overall, 18 out of 21 tested diseases were statistically significantly associated with PIM use. Parkinson's disease, arterial hypertension, epilepsy and depression were the most important factors associated with PIM use (OR ≥ 2), with 16.2‐, 4.5‐, 4.3‐ and 2.1‐fold increased odds, respectively. Acute coronary syndromes (39% lower odds) were the only morbidities that were inversely associated with PIM use.

### Frequency of and factors associated with medication underuse

We identified 10,371 (19.1%) participants with underuse.

The results from the age, sex and study centre–adjusted logistic regression model for the potential factors associated with underuse are presented in Table S6. In the full model (Table 3), males had 8% higher odds for underuse compared to females. Compared to those aged 60–64 years, participants aged 65–69 and ≥70 years had 16% and 25% lower odds for medication underuse, respectively. Participants in 9 out of 21 study centres had statistically significantly lower odds for underuse compared to participants attending the study centre in Augsburg. The lowest odds for underuse were observed in Waren (Müritz) (49% lower odds).

In contrast to other lifestyle factors, only obesity was statistically significantly associated with underuse, with 18% lower odds for underuse among obese participants compared to those with a normal BMI.

We detected 12 out of the 21 tested diseases as being statistically significantly associated with underuse. Thereof, osteoporosis (9.5‐fold increased odds), atrial fibrillation (6.1‐fold increased odds), Parkinson's disease (2.6‐fold increased odds) and chronic obstructive pulmonary disease (COPD) (2.0‐fold increased odds) were identified as particularly strongly associated with underuse (OR ≥ 2). The ranked proportions of participants with underuse per indication are presented in Table 4. We observed that more than half of the participants with osteoporosis (51.7%) and more than a third (38.7%) of the participants with atrial fibrillation had medication underuse for the specific indication. Parkinson's disease ranked fourth with 15.7% and COPD ranked sixth with 11.2% of patients with underuse.

**Table 4:** Ranking of proportion of participants with underuse of medication per indication in NAKO participants aged 60–74 years (N = 54,296).

Indication	*N*_indication_	*N* (%) with underuse for specific indication
Osteoporosis	3955 (7.3)	2046 (51.7)
Atrial fibrillation/Arrhythmia	8235 (15.2)	3187 (38.7)
Heart failure	2976 (5.5)	498 (16.7)
Parkinson's disease	197 (0.4)	31 (15.7)
Stroke	1879 (3.5)	267 (14.2)
COPD	4069 (7.5)	457 (11.2)
Arterial hypertension	26,961 (49.7)	1990 (7.4)
Gastrointestinal symptoms^a^	16,540 (30.5)	1110 (6.7)
Acute coronary syndromes	4391 (8.1)	265 (6.0)
Diabetes	6698 (12.3)	336 (5.0)
Cognitive impairment/Dementia^b^	2234 (4.1)	105 (4.7)
Myocardial infarction	2234 (4.1)	103 (4.6)
Pain^c^	36,887 (67.9)	1620 (4.4)
History of depression^d^	8531 (15.7)	89 (1.0)^e^
Thyroid disease/Hypothyroidism^f^	14,951 (27.5)	12 (0.1)
Epilepsy	396 (0.7)	0 (0.0)

*Note*: Exposure was defined according to the ‘Fit fOR The Aged’ (FORTA) list 2024.

Abbreviation: COPD, chronic obstructive pulmonary disease.

^a^ Combination of self‐reported information on reflux, peptic ulcer ('Have you ever been diagnosed with this condition by a physician?') and regular intake of NSAIDs (ATC M01A). Underuse was only considered if the participant was diagnosed with reflux or peptic ulcer in the year of their baseline assessment and in participants regularly taking NSAIDs.

^b^ Based on the interview question ‘Have you ever contacted a physician due to memory difficulties or are you planning to speak to a physician about it?’.

^c^ Pain‐related diagnoses included certain or likely migraine or tension headache in the last 12 months (following the third edition of The International Classification of Headache Disorders), and self‐reported information on arthritis, osteoarthritis and gout.

^d^ Lifetime history of depression, self‐reported.

^e^ Underuse was only considered in untreated individuals with severe depression according to the patient health questionnaire (PHQ‐9, sum score ≥20).

^f^ Lifetime history of thyroid disease, self‐reported. Underuse was only considered in individuals with TSH > 8.0 mU/L (cut‐off for treatment of older patients suggested by FORTA list).

Conversely, a lifetime history of an acute coronary syndrome and renal impairment were inversely associated with underuse, with 33% and 25% lower odds.

### Frequency of and factors associated with medication overuse

Medication overuse was detected in 12,787 (23.6%) participants. The most frequently overused drug classes are ranked in Table 5. No indication for low‐dose acetylsalicylic acid (ASA), hormone replacement therapy (HRT), proton pump inhibitors (PPIs) and antidepressants use could be found in 5.1%, 3.6%, 3.3% and 2.2% of the total study population, respectively. All other medication classes were identified as being overused in less than 2% of the total study population.

**Table 5:** Ranking of medication classes classified as overuse in NAKO study participants aged 60–74 years (N = 54,296).

	*N* (%)
Medication	Among total study population (*N* = 54,296)
ASA, low‐dose	2769 (5.1)
Hormone replacement therapy	1953 (3.6)
Proton‐pump inhibitors	1799 (3.3)
Antidepressants	1195 (2.2)
Antidementia drugs^a^	774 (1.4)
Antihypertensives	785 (1.4)
Anticoagulants	725 (1.3)
Beta blockers	531 (1.0)
Drugs used in obstructive airway diseases^b^	531 (1.0)
Ginkgo and ginseng preparations	519 (1.0)
Expectorants, mucolytics^c^	485 (0.9)
Benzodiazepines, *z*‐drugs	467 (0.9)
Levothyroxine	455 (0.8)
Antiparkinson medications	440 (0.8)
Systemic glucocorticoids	390 (0.7)
Diuretics	338 (0.6)
Statins	264 (0.5)
Antipsychotics	171 (0.3)
Sedative antihistamines	167 (0.3)
NSAIDs for long‐term use	154 (0.3)
Paracetamol	143 (0.3)
Antiplatelet drugs except ASA	130 (0.2)
Antiepileptics	125 (0.2)
Antidiabetics	119 (0.2)
Metamizole	117 (0.2)
Antiarrhythmics	77 (0.1)
Nitrates^d^	73 (0.1)
Opioids	72 (0.1)
Bisphosphonates	66 (0.1)
Lipid modifying agents except statins	64 (0.1)
H2 receptor antagonists	43 (0.1)
Other	68 (0.1)

*Note*: Exposure was defined according to the ‘Fit fOR The Aged’ (FORTA) list 2024.

Abbreviations: ASA, acetylsalicylic acid; NSAIDs, non‐steroidal anti‐inflammatory drugs.

^a^ Excluding ginkgo and ginseng preparations.

^b^ Including theophylline, roflumilast, inhalative corticosteroids, inhalative anticholinergics and inhalative beta 2 mimetic agents.

^c^ Including mucolytic agents and antitussives.

^d^ Including molsidomine.

For the factors associated with overuse, the results from the age, sex and study centre–adjusted logistic regression models are provided in Table S6. In the fully adjusted model (Table 3), we observed 25% lower odds for overuse in males compared to females, and 21% and 46% increased odds in participants aged 65–69 and ≥70 years compared to those aged 60–64 years. Furthermore, six study centres were statistically significantly associated with overuse, ranging from statistically significantly 22% lower odds for overuse in Bremen to 21% increased odds in Regensburg compared to the study centre in Augsburg. No other socio‐economic/‐demographic factors were statistically significantly associated with overuse after adjustment for multiple testing.

All assessed lifestyle factors were associated with overuse. Obesity and alcohol abstinence, as well as current and ≤20 years since quitting smoking, were positively associated with overuse, whereas sufficient physical activity was inversely associated with it.

After adjustment for multiple testing, 12 out of the 21 tested diseases were statistically significantly associated with overuse. Thereof, a lifetime history of depression (1.6‐fold increased odds) and epilepsy (1.5‐fold increased odds) were the factors exhibiting the strongest association (OR ≥ 1.5). Conversely, atrial fibrillation, acute coronary syndromes and pain were inversely associated with overuse, with 18%, 14% and 9% lower odds for overuse.

### Frequency of vaccination underuse

In the subsample with complete available information on administered vaccinations, 90.6% and 62.7% of study participants did not have the recommended pneumococcal and annual influenza virus vaccinations, respectively.

## Discussion

### Summary of findings

Our study summarized real‐world data of *n* = 54,296 participants aged 60–74 from the NAKO and showed that PIM use, underuse and overuse of medications according to the FORTA list [20] were very common, with 26.1%, 19.1% and 23.6% exposed individuals. Furthermore, we observed 90.6% and 62.7% underuse with respect to the recommended pneumococcal and annual influenza virus vaccinations in participants with complete available vaccination information, respectively. In addition, we identified multiple factors associated with PIM use, overuse and underuse in the areas of sociodemographic/‐economic factors, lifestyle factors and comorbidities.

### Comparison of frequencies of FORTA‐based medication quality estimates with those from previous studies

Multiple other, explicit medication assessment tools aim at improving optimization of pharmacotherapy for geriatric patients [18, 19]. Given that they address different aspects of medication quality, we will not go into details about studies utilizing other lists.

Despite the application of the FORTA list in previous studies [7, 33, 34, 35, 36, 37, 38, 39, 40, 41, 42], our study is the first to assess the frequency of all three subdomains of suboptimal medication quality, including PIM use, underuse and overuse in the general older population.

Proportions reported by previous studies [5, 7, 33, 34, 35, 36, 37, 38, 39, 40, 41, 42, 43, 44, 45] ranged from 52.6% to 76.5% [5, 42, 44, 45] for PIM use and from 48.7% [5] to 62.8% [44] for underuse. Only one study [5] reported a prevalence for overuse, and two out of three participants (66.7%) were exposed.

Although all these studies included participants with a mean age of 74 years or higher, our study population was relatively young (median age: 65 years). Furthermore, the morbidity was lower in the population‐based NAKO sample compared to the high‐risk populations included in the previous studies. Hence, it did not surprise that all previous studies reported higher proportions of exposed individuals [5, 7, 33, 34, 35, 36, 37, 38, 39, 40, 41, 42, 43, 44, 45]. In addition, a healthy volunteer bias is apparent in the NAKO study population, which precludes the estimation of disease or medication use prevalences for the German population without using weights. We, therefore, only report frequencies rather than prevalences and recommend treating them with caution. However, although our observations likely underestimated the prevalences of PIM use, medication underuse and overuse in the ‘true’ older general German population, they are still quite high, calling for measures to reduce them.

### Factors associated with PIM use

Since no previous study assessed factors associated with PIM use based on the FORTA list, we cautiously refer to studies that applied other tools [8, 9, 10, 22, 23]. Previous research found inconsistent results on potential associations of PIM intake with sex and age [8, 9, 10, 22, 23]. Our observation that older age and female sex were statistically significantly associated with PIM use is supported by three previous studies [8, 9, 23].

The higher risk of PIM use among individuals with low income, with obesity, insufficient physical activity, alcohol abstainers and current smokers has not yet been shown by previous German studies [9, 10], but they align with international research findings [46, 47, 48], suggesting that individuals with lower socio‐economic status and less healthy lifestyles might be more likely to receive PIM. Abstinence from alcohol might be seen as an indicator for a healthy lifestyle. However, in population‐based studies, abstainers are usually less healthy than low alcohol consumers, as they also include previous alcoholics and individuals who quit drinking due to diseases or interactions with their medication [49, 50].

It was not surprising that the vast majority of assessed diseases (18 out of 21) were statistically significantly associated with PIM use, as most of them are also FORTA indications. All cardiovascular diseases were among these factors, but arterial hypertension stood out with a particularly high 4.5‐fold increased odds for PIM use. However, all second‐ and third‐line antihypertensives are categorized as FORTA C or D [20]. Hence, simply switching these medications to potentially more appropriate alternatives is not feasible in many cases and may partly explain the high PIM rate for this indication.

We also identified three neurological diseases among the factors with the strongest associations with PIM use: Parkinson's disease, epilepsy and depression, with 16.2‐, 4.3‐ and 2.1‐fold increased odds for PIM use. The varying response rates and pharmacologic profiles of antiparkinson medications, antiepileptics and antidepressants are likely the challenging aspects for optimizing the indication‐specific therapy. For example, it would be essential to consider the differing pharmacological profiles of antidepressants, as all classes, except selective serotonin reuptake inhibitors, are classified as PIMs according to the FORTA list [20].

### Factors associated with underuse

Previous studies assessing factors associated with underuse have been based on guidelines for specific indications [25, 51, 52] or the START criteria [4]. Our finding that older age groups had higher odds of medication underuse resulted from the comprehensive adjustment for diseases. In the model adjusted for age, sex and study centre, only, no association between age and underuse was detected. This would be in line with a previous study from Germany, which found no association between age and underuse [4]. The observed 8% higher odds for underuse among males compared to females might be explained by sex differences in symptom awareness, preventive health behaviours and the subsequent number of physician contacts [53].

Obesity was the only lifestyle factor exhibiting an inverse association with underuse in the full model. This was also not observed in the age, sex and study centre–adjusted model and appeared after adjustment for the diseases. Maybe individuals with obesity as a comorbidity have more physician contacts and get more drugs prescribed.

Regarding the diseases, osteoporosis, atrial fibrillation, Parkinson's disease and COPD appeared to be most relevant, as they were associated with more than 2.0‐fold increased odds for underuse. Over half (51.7%) of the participants with osteoporosis did not take the indicated medication, which aligns with previous studies [34, 36, 41]. Given that osteoporosis‐related hip fractures are associated with higher mortality [54, 55] and economic burden [54, 56], undertreatment of osteoporosis has been recognized as an important national [57, 58, 59, 60] and global [57, 58, 61, 62, 63, 64] concern. As FORTA‐based prescribing has been proven to statistically significantly reduce underuse of osteoporosis medications in two randomized controlled trials [34, 36], there is sufficient evidence to implement such interventions into standard care.

The observed 6.1‐fold increased odds for underuse among participants with atrial fibrillation points to another area that requires clinical attention. Although we could not evaluate previous successful treatments through cardioversion or ablation, the 38.7% of medication underuse in patients with atrial fibrillation aligns with the 19% to over 50% underuse of anticoagulation among high‐risk patients identified by previous studies [51, 52, 65]. However, given the increasing prevalence of atrial fibrillation with age, the complexity of bleeding risks, optimal stroke prevention and rhythm management in the oldest patient group [66, 67], the observed high medication underuse rate should receive attention in clinical care.

For some diseases with considerable indication‐specific underuse rates, for example, Parkinson's disease and COPD, lack of efficacy of the first‐line drugs or early‐stage diseases, for which the therapy had not yet been initiated, could be an explanation for underuse.

### Factors associated with overuse

We only identified one previous study assessing factors associated with overuse, and this study only focused on antidepressants based on the START criteria [25]. We found 25% lower odds for overuse among men compared to women. This sex difference was likely driven by HRT, ranking second among all overused medication classes. The FORTA list classifies HRT as overuse for subjects without osteoporosis if it is not used within 3 years of menopause [2, 20], which typically occurs between the ages of 50 and 51 in Europe [68]. HRT was subsequently classified as overuse in all study participants without osteoporosis, since our study population was 60 years and older. Periodically re‐evaluating the need for HRT and the individual risk profile is also recommended by the European clinical practice guideline [69].

Regarding lifestyle factors, the clear pattern emerged that participants leading an unhealthy lifestyle appeared to be more likely to overuse medications. Maybe physicians tend to treat these patients with drugs rather than recommending lifestyle changes, which could be overuse if there is no clear indication. Furthermore, patients with an unhealthy lifestyle are often the most morbid and often receive polypharmacy. As the number of diseases increases, the medication gets more complex, and it is harder for the physicians to maintain an overview of all drugs (still) needed.

Diseases were less prominent factors associated with overuse compared to PIM use and underuse, but history of depression and epilepsy (1.6‐ and 1.5‐fold increased odds) appeared to be most relevant. A large proportion of overuse among patients with depression can be explained by maintenance therapy or relapse prophylaxis [70, 71, 72, 73] because 11.2% of participants with a lifetime history of depression used antidepressants but reported no moderate or severe symptoms, which we applied to define an indication for antidepressants. As previous research recommended maintenance therapy for at least 6 months after remission to effectively prevent relapse [72, 73], the absence of at least moderate depressive symptoms could also reflect the effectiveness of an appropriate and indicated treatment. Distinguishing ‘true’ overuse from appropriate maintenance therapy requires evaluating each individual's treatment regularly in clinical practice. However, we had no information on whether these assessments took place. Antidepressants and antiepileptics used to treat pain could not have been a driver of overuse since pain was considered an alternative indication.

When further exploring the underlying sources of overuse among patients with a history of depression or epilepsy, we found considerable overuse of low‐dose ASA and PPIs among participants with these indications. With respect to epilepsy, the high proportions of low‐dose ASA overuse might result from appropriate secondary stroke prevention, as stroke is the most common cause of epilepsy in older individuals [74]. If the history of stroke remained unreported, this resulted in overuse. Additionally, PPI might have been initiated due to gastrointestinal symptoms linked to depression [75] or caused by antiepileptic drugs [76]. If these symptoms were not severe, the drugs to treat them appear to be an overuse. Regarding low‐dose ASA and depression, associations between cardiovascular symptoms and depression have also been established [77]. However, if no cardiovascular disease has been diagnosed yet, the low‐dose ASA appears as overuse.

Our finding that low‐dose ASA was the most frequently overused medication (in 5.1% of the study population) should also receive attention in clinical practice. This frequency was in agreement with an observational study from Spain, which found overuse of low‐dose ASA in 7% of the participants [78]. Given the associated risks of bleeding, current guidelines have become very conservative regarding its use in primary prevention [79, 80]. The US guidelines even recommend against initiating low‐dose ASA for primary prevention in adults over 60 years of age [80].

In addition to the identified associated factors, an interesting finding was that 3.3% of the study population overused PPI. Concerns about their appropriate use have been progressively highlighted and addressed by specific deprescribing guidelines [81, 82]. A previous drug utilization study of German claims data revealed a notable rise in PPI prescriptions between 2010 and 2016, and 52% of new PPI users had no documented on‐label indication [83].

### Underuse of vaccinations

The low vaccination rates for pneumococcal (90.6% underuse) and annual influenza (62.7% underuse) vaccinations observed in our study align with recent data from the German vaccination monitoring: 88.2% and 68.6% of the citizens aged 60–64 and 65–69 years, respectively, had not received pneumococcal vaccinations. Furthermore, 61.8% of those aged 60 years and older had no influenza vaccination [84]. Implementing regular vaccination checks, ideally through easily accessible opportunities outside of standard physician visits, could help reduce the underutilization of vaccines.

### Regional differences

Regional social inequalities between rural and urban German regions have been previously recognized, highlighting better accessibility to primary and specialist care in urban regions [85, 86, 87]. Our findings might reflect these disparities by showing differences in odds for all three subdomains of medication quality by study centre. For instance, the odds for PIM use were considerably low in Hamburg, which is the second largest town in Germany and has a higher average socio‐economic status and a higher ratio of physicians per inhabitant compared to most other German rural regions. Consequently, participants from study centres in rural eastern German regions, that is, Neubrandenburg and Demmin, had the highest odds for PIM use. Interestingly, these rural regions also had the lowest odds for underuse. With respect to overuse, the pattern was less clear, as urban regions were among the study centres with the highest (Regensburg) and lowest (Bremen) odds for overuse. We consider these potential regional differences interesting signals warranting further investigation.

### Strengths and limitations

To our knowledge, this is the first study investigating the frequency of and factors associated with all three dimensions of suboptimal prescribing in the general older population. The availability of a wealth of data enabled us to assess potential associations of a broad range of factors. Incorporating blood‐based biomarkers as well as diagnostic and physical assessments in our indication assessment is a unique strength that helped minimize potential misclassification for diagnoses. Moreover, the comprehensive brown‐bag review ensured a high level of reliability and completeness of the medication assessment. Finally, the high sample size (*n* = 54,296) provided sufficient statistical power to address a large number of potentially associated factors (*n* = 31) with correction for multiple testing. Although the identified associated factors provide a clinically relevant foundation for effectively identifying target populations for medication reviews, it should be noted that the cross‐sectional design mainly reflects correlations but does not imply causal inference.

The main limitation of our study is the ‘healthy volunteer’ bias and a relatively young sample of older adults (60–74 years). Hence, our results cannot be extrapolated to older populations or all German adults aged 60 years and older.

Another important limitation is the risk of information bias, including both over‐ and underreporting of indications (e.g., through recall bias or undiagnosed conditions) and (over‐the‐counter) medications. We addressed this by comprehensive statistical adjustment, completing indications with information from medications and biomarkers.

We used the 2024 version of the FORTA list, whereas data collection was from 2014 to 2019. We think that the minimal changes during this time did not affect our results to a relevant extent. However, revisions and expansions of the FORTA list over time may influence prevalence estimates and complicate comparisons across studies using different versions. Our German study population and the use of the Germany‐specific FORTA list restrict the generalizability to other countries. However, a European version of the FORTA list is also available [88] that largely aligns with the Germany‐specific version. A future study across European countries, preferably with available linkage to validated diagnoses (e.g., claims data), would be desirable.

### Clinical implications

The results highlight critical areas, which clinical practice may need to address, such as untreated osteoporosis and low vaccination rates. In addition, the factors associated with the three subdomains of suboptimal medication can be used to assign resources for comprehensive medication reviews with the FORTA list to those patients with the highest odds for PIM use, underuse or overuse, which were patients with arterial hypertension, Parkinson's disease, epilepsy, depression, osteoporosis, COPD and atrial fibrillation. As arterial hypertension has a very high prevalence in older adults (49.7% in our sample), and all second‐ and third‐line antihypertensives are categorized as PIM, making changes in antihypertensive medication difficult, we recommend focusing on the other named, less common, six indications. As ‘only’ 0.4%, 0.7%, 15.7%, 7.3%, 7.5% and 15.2% of the older German NAKO study population were affected by Parkinson's disease, epilepsy, depression, osteoporosis, COPD and atrial fibrillation, respectively, and as they are also usually documented in electronic health records, we suggest that they could be used to identify a high‐risk population for suboptimal medication quality quickly. If resources for comprehensive medication reviews with the FORTA list are limited, this disease‐defined high‐risk population could be prioritized. However, it should be noted that this recommendation is not based on evidence from RCTs showing a higher benefit from comprehensive medication reviews in these patient populations compared to others. Such RCTs are still needed. Moreover, further research could establish effective strategies that help clinicians address suboptimal medication quality in hard‐to‐reach groups, such as patients with lower socio‐economic status, leading unhealthy lifestyles or living in rural regions with barriers to healthcare access. Finally, the use of the FORTA list should only be regarded as a screening tool to detect suboptimal medication. It cannot replace clinical judgement, and the ultimate decisions regarding (de‐)prescribing should also be guided by individual patient goals and preferences.

## Conclusion

PIM use, medication underuse and overuse were very common in this large, population‐based study of older adults from Germany. Arterial hypertension, Parkinson's disease, epilepsy, depression, osteoporosis, COPD and atrial fibrillation were identified as diseases with the highest odds of insufficient medication quality. If resources for medication reviews are limited, the latter six diseases could be prioritized because they have prevalences <16%.

## Author contributions

*Conceptualization*: Miriam Degen and Ben Schöttker. *Methodology*: Miriam Degen and Ben Schöttker. *Resources*: Miriam Degen, Margit Heier and Jakob Linseisen. *Data preparation*: Miriam Degen. *Formal analysis*: Miriam Degen. *Preparation of the manuscript*: Miriam Degen. *Review and editing*: all authors. *Supervision*: Ben Schöttker. All co‐authors accounted for reviewing and editing of the manuscript. Ben Schöttker and Miriam Degen are accountable for all aspects of the work in ensuring that questions related to the accuracy or integrity of any part of the work are appropriately investigated and resolved. All co‐authors accounted for reviewing and editing of the manuscript. Ben Schöttker and Miriam Degen are accountable for all aspects of the work in ensuring that questions related to the accuracy or integrity of any part of the work are appropriately investigated and resolved.

## Conflict of interest statement

The authors declare no conflicts of interest.

## Funding information

This work was supported by the German Cancer Aid, Grant/Award Number: 70115079; German National Cohort (NAKO), application number: NAKO‐960; Federal Ministry of Research, Technology and Space (BMFTR), project funding reference numbers: 01ER1301A/B/C, 01ER1511D, 01ER1801A/B/C/D, 01ER2301A/B/C; federal states of Germany; Helmholtz Association; participating universities; and Leibniz Association Institutes.

## Supporting information




**Text S1**: Detailed methods used for multiple imputation.
**Figure S1**: Flow chart of the study population.
**Table S1**: Definitions of FORTA indications.
**Table S2**: FORTA classification and ATC codes of assessed medications.
**Table S3**: Comparison of baseline characteristics of study participants included in the subsample with complete vaccination information (*N*=5,575) and those excluded from it (*N*=48,721).
**Table S4**: Percentages of missing data of covariates prior to imputation in population analyzed (*N*=54,296).
**Table S5**: Percentages of missing data of indications for medication use prior to imputation in population analyzed (*N*=54,296).
**Table S6**: Potential factors associated with PIM, medication underuse, and overuse in the NAKO study ‐ Results of the logistic regression model adjusted for age, sex, and study centre (*N*=54,296).

## Data Availability

Data are available upon reasonable request and require prior approval at the NAKO and a signed data transfer agreement (DTA) with the NAKO.
