# Supplementary material for: Factors associated with potentially inappropriate medication use, medication underuse and overuse in older adults in the German National Cohort
Source: J Intern Med. 2026 Apr 11;299(6):754–75. doi: 10.1111/joim.70097 (PMC13137406; doi:10.1111/joim.70097)
Supplement: Supplementary file 1 — Text S1: Detailed methods used for multiple imputation. Figure S1: Flow chart of the study population. Table S1: Definitions of FORTA indications. Table S2: FORTA classification and ATC codes of assessed medications. Table S3: Comparison of baseline characteristics of study participants included in the subsample with complete vaccination information (N=5,575) and those excluded from it (N=48,721). Table S4: Percentages of missing data of covariates prior to imputation in population analyzed (N=54,296). Table S5: Percentages of missing data of indications for medication use prior to imputation in population analyzed (N=54,296). Table S6: Potential factors associated with PIM, medication underuse, and overuse in the NAKO study ‐ Results of the logistic regression model adjusted for age, sex, and study centre (N=54,296). [file JOIM-299-754-s001.docx]

**Supplementary Material to Article**

“Factors associated with potentially inappropriate medication use, medication underuse, and overuse in older adults in the German National Cohort”

Table of contents

[Text S1. Detailed methods used for multiple imputation 2](#_Toc223096600)

[Figure S1. Flow chart of the study population 3](#_Toc223096601)

[Table S1. Definitions of FORTA indications 4](#_Toc223096602)

[Table S2. FORTA classification and ATC codes of assessed medications 6](#_Toc223096603)

[Table S3. Comparison of baseline characteristics of study participants included in the subsample with complete vaccination information (N=5,575) and those excluded from it (N=48,721) 13](#_Toc223096604)

[Table S4. Percentages of missing data of covariates prior to imputation in population analyzed (N=54,296) 17](#_Toc223096605)

[Table S5. Percentages of missing data of indications for medication use prior to imputation in population analyzed (N=54,296) 18](#_Toc223096606)

[Table S6. Potential factors associated with PIM, medication underuse, and overuse in the NAKO study - Results of the logistic regression model adjusted for age, sex, and study centre (N=54,296) 19](#_Toc223096607)

[References 23](#_Toc223096608)

# Text S1. Detailed methods used for multiple imputation

We explored missing patterns visually using a grid display, yielding an arbitrary missing pattern, and potential sources of deviations from a missing completely at random (MCAR) occurrence. Applying the SAS procedure ‘PROC MI’ with the fully conditional specification (FCS) algorithm [1] with 20 burn-in iterations, we subsequently used an expanded imputation model under the missing at random (MAR) assumption that explicitly incorporated identified sources for non-MCAR (sex, income, and grip strength) and additional auxiliary variables (e.g., high density lipoprotein blood levels as proxy for metabolic health) to mitigate potential risks of bias due to deviations from MCAR.

Specifically, the following variables were included in the imputation model: age, sex, study centre, income, years of education, social network index, body-mass index, daily alcohol consumption, smoking status and age at smoking cessation, self-perceived general health status, fractures since aged 50 years, pain in last four weeks, headache, gout, arthritis, osteoarthritis, self-reported lifetime history of depression, stroke, coronary heart disease, myocardial infarction, asthma, chronic obstructive lung disease, peripheral vascular disease, lifetime history of cancer, heart failure, atrial fibrillation, cardiac arrhythmia, epilepsy, Parkinson’s disease, self-perceived memory difficulties, planned or prior physician consultation for cognitive impairment, reflux, peptic ulcer, insomnia, osteoporosis, diabetes, hypertension, high density lipoprotein, creatinine, haemoglobin A_1c_, systolic blood pressure, diastolic blood pressure, grip strength.

Goodness of fit of the imputation model was thoroughly examined through trace plots and comparison of distributions before and after imputation in line with recommended practice [2, 3], indicating adequate model performance.

To derive analytical outcomes, we aggregated the imputed datasets using the SAS procedure ‘PROC MIANALYZE’


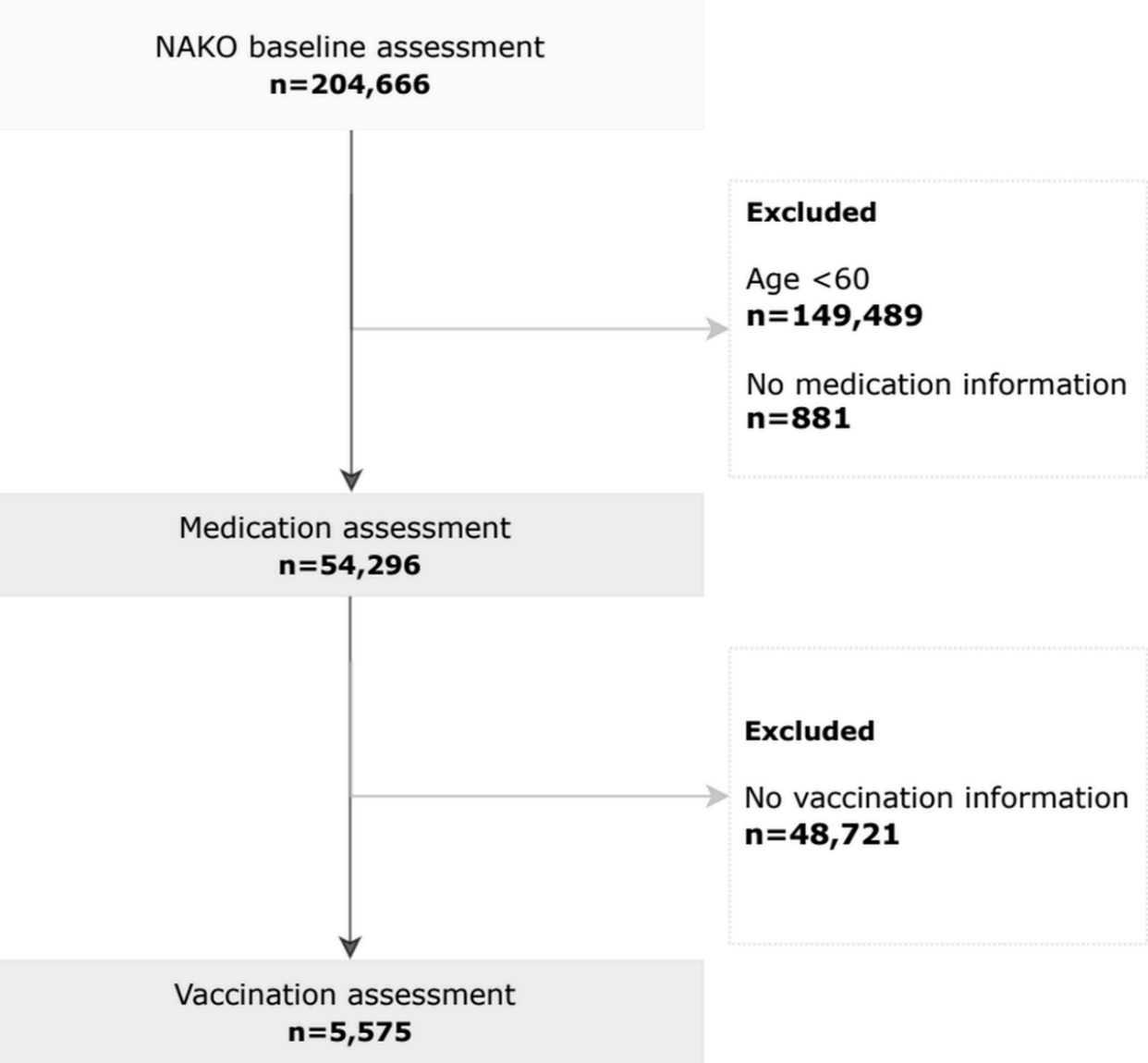


# Figure S1. Flow chart of the study population

# Table S1. Definitions of FORTA indications

| **FORTA Indication** | **Description** | **Additional information** |
| --- | --- | --- |
| Diabetes | Combination of self-reported information and use of diabetes medication (ATC A10), use of oral antidiabetics and/or GLP-1-RAs (ATC A10B, A10X), and use of insulin (ATC A10A). Categories: No, untreated, treated with oral antidiabetics, treated with insulin. | Underuse was only considered in untreated individuals with HbA1c > 48 mmol/mol |
| Arterial hypertension | Combination of self-reported information and use of antihypertensives (ATC C02, C03, C04, C08CA, C09) | Underuse was only considered in untreated individuals with systolic blood pressure > 140 mmHg |
| Atrial fibrillation | S.r. | Note: interview question covered any type of cardiac arrythmia and was not specific to atrial fibrillation |
| Stroke | S.r. |  |
| Myocardial infarction | S.r. |  |
| Heart failure | S.r. |  |
| Acute coronary syndromes | Combination of self-reported information on history of coronary heart disease and myocardial infarction |  |
| COPD | S.r. | Note: also included chronic bronchitis. Underuse was only considered in individuals with Tiffenau Index < 0.7 |
| Osteoporosis | S.r. |  |
| Thyroid disease/ hypothyroidism | S.r. | Interview question covered any thyroid disease and was not specific to hypothyroidism. Underuse was only considered in individuals with TSH > 8.0 mU/L (cut-off for treatment of older patients suggested by FORTA list) |
| Pain | Combination of self-reported moderate, severe, or very severe pain during the last 4 weeks (‘How severe has your pain been in the past 4 weeks?’) and pain-related diagnoses. Pain-related diagnoses included certain or likely migraine or tension headache in the last 12 months (following the 3^rd^ edition of The International Classification of Headache Disorders), and self-reported information on arthritis, osteoarthritis, and gout. |  |
| Parkinson’s disease | S.r. |  |
| Cognitive impairment/dementia | Subjective cognitive impairment, combination of interview questions: ‘How would you evaluate your memory at the moment?’ – Five categories: very good to very bad; and: ‘Have you ever talked to a physician about your memory difficulties, or do you plan to talk to a physician about this?’ | The full model was adjusted for a binary variable indicating bad or very bad subjective cognitive impairment. The same variable was used as diagnosis in the PIM assessment; underuse was only considered in untreated individuals indicating having the worst category of memory; overuse was not considered in individuals that indicated very bad or bad memory or that indicated moderate memory and consulted a physician or planned to consult a physician due to memory issues |
| Depression | Combination of self-reported information (‘Have you ever been diagnosed with this condition by a physician?’) and PHQ-9 sum score (0-14: no or mild depression, 15-19: moderate to severe depression, ≥ 20: severe depression) | Self-reported lifetime history of depression was used for statistical adjustment;  PIM: self-reported depression or moderate to severe depression according to PHQ-9 (sum score ≥ 15); overuse assessment: moderate to severe depression according to PHQ-9 (sum score ≥ 15);  underuse was only considered in untreated individuals with severe depression according to or PHQ-9 (sum score ≥ 20). |
| Epilepsy | Classified as certain, possible, or no diagnosis, based on an algorithm created by the NAKO expert group neurological and psychiatric disorders, considering self-reported information on ever diagnosis and time since first diagnosis, treatment during the last 12 months, and current use of antiepileptics. | The model was adjusted for a binary variable (no/certain or possible);  PIM assessment: considered possible and certain diagnosis;  underuse was only considered in untreated individuals with certain diagnosis;  overuse was not considered in participants with possible or certain diagnosis |
| Insomnia | Difficulties falling asleep ≥ 3 days per week or difficulty staying asleep ≥ 3 times per week | Underuse was not assessed due to the large proportion (94.7%) of individuals with underuse for this indication |
| Gastrointestinal symptoms | Combination of self-reported information on reflux, peptic ulcer (‘Have you ever been diagnosed with this condition by a physician?’), and regular intake of NSAIDs (ATC M01A) | Underuse was only considered if the participant was diagnosed with reflux or peptic ulcer in the year of their baseline assessment and in participants regularly taking NSAIDs |

Abbreviations: ATC, Anatomical Therapeutic Chemical Classification; COPD, chronic obstructive pulmonary disease; FORTA, Fit for the aged; GLP-1-RA, Glucagon-like peptide-1 receptor agonists; HbA1c, haemoglobin A1c; NSAIDs, non-steroidal anti-inflammatory drugs; PHQ-9, patient health questionnaire; PIM, potentially inappropriate medication; s.r., self-reported previous diagnosis; TSH, thyroid stimulating hormone.

# Table S2. FORTA classification and ATC codes of assessed medications

| **Medication** | **Class^a^** | **ATC codes^b^** |
| --- | --- | --- |
| ***Arterial hypertension*** |  |  |
| ACE-inhibitors,  angiotensin receptor antagonists | A | C09A, C09B, C09C, C09D, C10BX04, C10BX06, C10BX07, C10BX11, C10BX12, C10BX10, C09DX04 |
| Long-acting calcium antagonists, dihydropyridine type | A | C08CA |
| Indapamide | A | C03BA11, C09BX01, C09BX06, C09BA54, C10BX13, C08GA22 |
| Other diuretics | B | C03 |
| *Hypertensives* | n.l. | *Summarized as C02, C03, C04, C05, C06, C07, C08, C09* |
| Alpha receptor antagonists | C | C02CA, C02LE |
| Spironolactone | C | C03DA01, C03EC41, C03ED01, C03EC21, C03EC01 |
| Moxonidine | C | C02AC05, C02LC05 |
| Aliskiren | C | C09XA02, C09XA53, C09XA52, C09XA54, C09DX02 |
| Betablockers^c^ | C | C07 |
| Atenolol^c^ | D | C07AB03, C07FX18, C07DB01, C07DB03, C07DB53, C07DB23, C07DB03, C07BB03 |
| Clonidin | D | C02AC01, C02LC01, C02LC51 |
| Minoxidil | D | C02DC01 |
| Calcium antagonists, verapamil type | D | C08D, C09BB10, C08GA53,  C08GA23 |
| ***Cardiac insufficiency*** |  |  |
| ACE-inhibitors, angiotensin receptor antagonists | A | C09A, C09B, C09C, C09D, C10BX04, C10BX06, C10BX07, C10BX11, C10BX12, C10BX10, C09DX04 |
| Betablockers | A | C07 |
| Diuretics | B | C03A, C03B, C03C, C03E, C03DB, C09BA, C09DX01, C09DX03, C09BX01 |
| Iron substitution | A | B03A |
| Sacubitril/Valsartan | A | C09DX04 |
| SGLT2 inhibitors | B | A10BK, A10BD15, A10BD16, A10BD19, A10BD20, A10BD21, A10BD23, A10BD24, A10BD25, A10BD27, A10BD29, A10BD30 |
| Mineralcorticoid antagonists | C | C03DA01, C03EC41, C03ED01, C03EC21, C03EC01, C03DA03, C03DA05, C03DA04 |
| Digitalis preparations | C | C01AA |
| Ivabradine | C | C01EB17, C07FX06, C07FX05 |
| ***Acute coronary syndromes*** |  |  |
| ACE-inhibitors, angiotensin receptor antagonists^d^ | A | C09A, C09B, C09C, C09D, C10BX04, C10BX06, C10BX07, C10BX11, C10BX12, C10BX10, C09DX04 |
| Low-dose ASA^e^ | A | B01AC34, B01AC36, C07FX03, C10BX02, B01AF51, C10BX05,  C10BX01, C10BX04, C07FX02, B01AC06, B01AC56, B01AC86, C10BX12, C10BX06, C10BX08, C10FX04 |
| Heparin | A | B01AB |
| Betablockers^f^ | A | C07 |
| Atorvastatin | A | C10AA05, C10BX08, C10BX03, C10BA05, C10BX12, C10BX06, C10BX11 |
| Nitroglycerin spray, acute single use on demand | A | C01DA02^g^ |
| Clopidogrel, prasugrel | A | B01AC04, B01AC22, B01AC34 |
| Thrombolytics, especially rTPA | B | B01AD |
| *Antithrombotic agents* | n.l. | *B01A* |
| Nitrates, long-acting | C | C01DA02,^g^ C01DA52,^g^ C01DA08,^g^ C01DA58,^g^ C01DA14, C01DA05, C01DA55 |
| Glycoprotein IIb/IIIa antagonist | C | B01AC13, B01AC16, B01AC17 |
| Ivabradine | C | C01EB17, C07FX06, C07FX05 |
| Molsidomine | D | C01DX12 |
| ***Myocardial infarction*** |  |  |
| ACE-inhibitors,  angiotensin receptor antagonists | A | C09A, C09B, C09C, C09D, C10BX04, C10BX06, C10BX07, C10BX11, C10BX12, C10BX10, C09DX04 |
| Low-dose ASA | A | B01AC06, C10BX08, C10BX12, C10BX06, C07FX04, C07FX03, C10BX02, C10BX05, C10BX01, C10BX04, C07FX02, B01AF51 |
| Clopidogrel | A | B01AC04, B01AC34 |
| Ezetimibe | B | C10AX09, C10BA05, C10BA06, C10BA02 |
| Statins | A, B | C10AA, C10BA, C10BX, A10BH51 |
| *Antithrombotic agents* | n.l. | *B01A* |
| Betablockers | B, C^f^ | C07 |
| Nitroglycerin spray | A, C^h^ | C01DA02^g^ |
| Bempedoic acid | C | n.a. |
| Fibrates | C | C10AB, C10BB02, C10BB01, C10BA03, C10BA04 |
| Ranolazine | C | C01EB18 |
| Amiodarone | C | C01BD01 |
| Other class-I/III antiarrhythmic agents | D | C01BA, C01BB, C01BC, C01BG, C01BD02, C01BD03, C01BD04, C01BD05, C01BD06, C01BD07, C07AA07, C07FX02, C07BA07 |
| Dihydropyridine antagonists^i^ | D | C08CA, C08GA, C10BX03, C10BX09, C10BX11 |
| Niacin | D | C10AD |
| PCSK9 inhibitors | D | C10AX13, C10AX14 |
| ***Stroke*** |  |  |
| Low-dose ASA | A | B01AC34, B01AC36, C07FX03, C10BX02, B01AF51, C10BX05,  C10BX01, C10BX04, C07FX02, B01AC06, B01AC56, B01AC86, C10BX12, C10BX06, C10BX08, C10FX04 |
| Statins | A | C10AA, C10BA, C10BX, A10BH51 |
| r-TPA | A | B01AD |
| Clopidogrel | A | B01AC04, B01AC34 |
| *Antithrombotic agents* | n.l. | B01A |
| Dipyridamole plus ASA | C | B01AC36 |
| ***Atrial fibrillation*** |  |  |
| Betablockers | A | C07 |
| NOACs | A, B^j^ | B01AE, B01AF |
| Warfarin | B | B01AA03 |
| Heparin^k^ | C | B01AB |
| Digoxin, digitoxin | C | C01AA |
| Phenprocoumon | C | B01AA04 |
| Amiodarone | C | C01BD01 |
| Other class I or III antiarrhythmic agents | D | C01BA, C01BB, C01BC, C01BG, C01BD02, C01BD03, C01BD04, C01BD05, C01BD06, C01BD07, C07AA07, C07FX02, C07BA07 |
| Diltiazem, verapamil | D | C08DB01, C08DA01, C09BB10, C08DA51, C08DA81, 08GA53, C08DA51, C08GA23 |
| Low-dose ASA^l^ | D | B01AC06, B01AC56, C10BX08, C10BX12, C10BX06, C07FX04, C07FX03, C10BX02, C10BX05, C10BX01, C10BX04, C07FX02 |
| ***COPD*** |  |  |
| Antibiotics except fluoroquinolones | A | Not assessed |
| Inhalative long-acting parasympatholytics | A | R03BB02, R03BB04, R03BB05, R03BB06, R03BB07, R03BB54, R03AL03, R03AL04, R03AL05, R03AL06, R03AL07, R03AL09 |
| Inhalative beta 2 mimetic agents | B | R03AC, R03AL, R03AK03, R03AK04, R03AK05, R03AK06, R03AK07, R03AK08, R03AK09, R03AK10, R03AK11, R03AK12, R03AK13 |
| Inhalative glucocorticoids | B | R03BA, R03AK06, R03AK07, R03AK08, R03AK09, R03AK10, R03AK11, R03AK12, R03AK13, R03AL09 |
| *Drugs for obstructive airway diseases* | n.l. | *Summarized as R03* |
| Systemic glucocorticoids | A, D^m^ | H02 |
| Mucolytic agents | C | R05CB |
| Roflumilast | C | R03DX07 |
| Theophylline | D | R03DA04, R03DB04, R03DA54, R03DA74 |
| Antitussives | D | R05DA, R05DB |
| ***Osteoporosis*** |  |  |
| Calcium and vitamin D^n^ | A | A12A, A11CC05, A11CC80 |
| Bisphosphonates | A, B | M05BA, M05BB |
| Raloxifene | A | G03XC01 |
| Denosumab | A | M05BX04 |
| Alfacalcidol | B, C^o^ | M05BB06, A11CC03 |
| Romosozumab | B | n.a. |
| Teriparatide | B | H05AA02 |
| Parathormone | C | H05AA03 |
| Nandrolone decanoate | D | A14AB01 |
| Fluoride | D | A12CD |
| Hormone replacement therapy | D | G03C, G03F, G03AA, G03AB |
| ***Diabetes*** |  |  |
| DPP-4 inhibitors | A | A10BH, A10BD07, A10BD08, A10BD09, A10BD10, A10BD11, A10BD12, A10BD13, A10BD18, A10BD19, A10BD21 |
| GLP-1-RAs | B | A10BJ |
| Insulin | B | A10A |
| Metformin | B | A10BA02, A10BD17, A10BD13, A10BD20, A10BD18, A10BD11, A10BD05, A10BD14, A10BD03, A10BD10, A10BD07, A10BD02, A10BD08, A10BD31, A10BD10 |
| SGLT2-inhibitors | B | A10BK, A10BD15, A10BD16, A10BD19, A10BD20, A10BD21, A10BD23, A10BD24, A10BD25, A10BD27 |
| Acarbose | C | A10BF01, A10BD17 |
| Glinides | C | A10BX02, A10BX03, A10BX08, A10BD14 |
| Sulfonylureas | C, D | A10BB, A10BD01, A10BD02, A10BD04, A10BD06 |
| PPARγ ligands | C, D | A10BG, A10BD09, A10BD12, A10BD04, A10BD03, A10BD05, A10BD06 |
| ***Cognitive impairment/dementia*** |  |  |
| Acetylcholinesterase inhibitors | B | N06DA |
| Memantine | B | N06DX01, N06DA52, N06DA53 |
| Ginkgo biloba | C | N06DA53, N06DP01 |
| Adacanumab | D | n.a. |
| Antioxidants: vitamin E, selenium, vitamin C | D | A11G, A11EB, A11HA03, A11CB, A12CE |
| Ginseng | D | A13AP02 |
| Hormone preparations | D | G03 |
| Lecanemab | D | n.a. |
| Piracetam | D | N06BX03 |
| Statins^p^ | D | C10AA, C10BA, C10BX, A10BH51 |
| ***Depression*** |  |  |
| SSRIs | B | N06AB |
| *Antidepressants* | n.l. | *Summarized as N06A* |
| Mirtazapine | C | N06AX11 |
| SNRIs | C | N06AX16, N06AX23, N06AX21, N06AX17 |
| Bupropion | C | N06AX12 |
| Quetiapine | C | N05AH04 |
| Tianeptin | C | N06AX14 |
| Benzodiazepines^q^ | C, D | N05CD, N05BA, N03AE |
| Agomelatine | D | N06AX22 |
| MAO-A inhibitors | D | N06AG |
| St. John’s Wort | D | N06AP01 |
| Reboxetine | D | N06AX18 |
| Trazodone | D | N06AX05 |
| Tricyclic antidepressants | D | N06AA, N06CA01 |
| ***Insomnia*** |  |  |
| Melatonin | B | N05CH01 |
| Daridorexant | C | n.a. |
| Z-drugs | C | N05CF |
| Pipamperone | C | N05AD05 |
| Melperone | C | N05AD03 |
| Mirtazapine | C | N06AX11 |
| Trazodone | C | N06AX05 |
| Doxepin | C, D | N06AA12 |
| Benzodiazepines^q^ | D | N05CD, N05BA, N03AE |
| Opipramol | D | N06AA05 |
| Sedative antihistamines | D | R06AA02, R06AA52, R06AC01, R06AC03, R06AC53, R06AX05, R06AC04, R06AA08, R06AA09, R06AA59, R06AA01, R06AA04, R06AA54, R06AA11, R06AB05, R06AB04, R06AB54, R06AB02, R06AB52, R06AB06, R06AB56, R06AB01, R06AB51, R06AX07, R06AE03, R06AE53, R06AE04, N05BB01, N05BB51, R06AE05, R06AE55, R06AD02, R06AD52, R06AD01, R06AX02, N05CM20, N05CM21, N05CM22 |
| ***Pain*** |  |  |
| Paracetamol | A | N02BE01, N02BE51, N02AJ06, N02AJ13, N02BE61, N02BE71 |
| Metamizole | B | N02BB02, N02BB52 |
| Opioids, e.g. Buprenorphine, oxycodone, hydromorphone | B | N02AE01, N02AA05, N02AA03 |
| *Analgesics* | n.l. | *Summarized as N02* |
| Morphine, fentanyl | C | N02AA01, N02AA51, N02AG01, N02AB03 |
| Opioid agonist/antagonist combinations | C | N02AX51, N02AA55 |
| SSRIs^r^ | C | N06AB |
| SNRIs | C | N06AX16, N06AX23, N06AX21, N06AX17 |
| Tapentadol | C | N02AX06 |
| Tramadol | C | N02AX02, N02AJ14, N02AJ15, N02AJ13 |
| Antiepileptics^s^ | C, D | N03AF02, N03AF04, N03AX15, N03AB02, N03AB52, N03AD01, N03AD51, N03AG01, N03AF01, N03AX12, N03AX16 |
| Tricyclic antidepressants | D | N06AA, N06CA01 |
| NSAIDs for long-term use^t^ | D | M01AA, M01AB, M01AC, M01AD, M01AE, M01BA01, M01BA03, N02BA51, N02BA71, N02BA01 |
| ***Epilepsy*** |  |  |
| Levetiracetam | B | N03AX14 |
| Lamotrigine | B | N03AX09 |
| Gabapentin | B | N03AX12 |
| Topiramat | B | N03AX11 |
| Lacosamide | B | N03AX18 |
| *Antiepileptics* | n.l. | *Summarized as N03A* |
| Lorazepam | B, D^t^ | N05BA06, N05BA56 |
| Pregabalin | C | N03AX16 |
| Valproic acid | C | N03AG01 |
| Carbamazepine | C | N03AF01 |
| Eslicarbamazepine | C | N03AF04 |
| Oxcarbamazepine | C | N03AF02 |
| Zonisamide | C | N03AX15 |
| Diazepam | C, D | N05BA01 |
| Midazolam | C, D | N05CD08 |
| Phenytoin | D | N03AB02, N03AB52 |
| Phenobarbital | D | N03AA02 |
| Ethosuximide | D | N03AD01, N03AD51 |
| ***Parkinsons disease*** |  |  |
| Levodopa | A | N04BA01, N04BA03, N04BA10, N04BA11, N04BA13, N04BA14 |
| COMT inhibitors | B | N04BX01, N04BX02, N04BA03, N04BX04 |
| Ropinirole | B | N04BC04 |
| Rotigotine | B | N04BC09 |
| *Anti-parkinson drugs* | n.l. | *Summarized as N04* |
| MAO-B inhibitors | C, D | N04BD02, N04BD01, N04BD03 |
| Pramipexole | C | N04BC05 |
| Piribedil | C | N04BC08 |
| Bromocriptine | D | N04BC01 |
| Cabergoline | D | N04BC06 |
| Amantadine | D | N04BB01 |
| Anticholinergics | D | N04A |
| ***Gastrointestinal symptoms*** |  |  |
| Proton pump inhibitors | B | A02BC |
| *Drugs for acid-related disorders* | n.l. | *Summarized as A02* |
| H_2_ receptor antagonists | C | A02BA |
| ***Hypothyroidism*** |  |  |
| L-Thyroxin | A^u^ | H03 |
| ***Vaccinations*** |  |  |
| Annual influenza vaccination | A | Interview question^v^ and vaccines administered according to the vaccination booklet |
| Pneumococcal vaccination | A | Administered vaccines according to the vaccination booklet |
| Herpes zoster vaccine | A | Not assessed |
| RSV vaccine | A | Not assessed |

Abbreviations: ACE, angiotensin-converting-enzyme; ASA, acetylsalicylic acid; ATC, Anatomical Therapeutic Chemical Classification; COMT, catechol-O-methyltransferase; COPD, chronic obstructive pulmonary disease; DPP-4, dipeptidyl peptidase-4; GLP-1-RA, Glucagon-like peptide-1 receptor agonists; MAO, monoamine oxidase; n.l., not listed in FORTA list; n.a., not available at time of baseline assessment; NOACs, new oral anticoagulants; NSAIDs, non-steroidal anti-inflammatory drugs; PCSK9, proprotein convertase subtilisin/kexin type 9; PPARγ, peroxisome proliferator-activated receptor gamma; RSV, respiratory syncytial virus ; rTPA, recombinant tissue plasminogen activator; SGLT2, sodium glucose linked transporter 2; SNRIs, serotonin-noradrenalin reuptake inhibitors; SSRIs, selective serotonin reuptake inhibitors.

^a^ Classes: A: indispensable; B: beneficial; C: questionable; D: avoid.
^b^ According to the German modification of ATC codes version 2018. ^c^ Only considered as PIM in participants without diagnosis of acute coronary syndrome, myocardial infarction, atrial fibrillation, and cardiac insufficiency.
^d^ FORTA only explicitly lists ACE inhibitors. Angiotensin receptor antagonists were also considered.
^e^ Not identified as overuse in participants with atrial fibrillation, peripheral vascular disease, history of myocardial infarction, stroke, angina pectoris, and other acute coronary syndromes.
^f^ In participants with a history of myocardial infarction, betablockers are only considered PIM if the date of the last myocardial infarction was >3 years ago and in the absence of other indications (atrial fibrillation, acute coronary syndromes, cardiac insufficiency).
^g^ Immediate-release glyceryl trinitrate formulations used on demand were not defined as PIM (e.g., spray, solution, bite capsules). Patches were identified as PIM.
^h^ A: on demand use. B: long-term use.
^i^ Only considered as PIM in participants without arterial hypertension.
^j^ A: Apixaban, B: other NOACs.
^k^ Not identified as PIM in participants with acute coronary syndromes.
^l^ Not identified as PIM in participants with myocardial infarction, acute coronary syndromes, or stroke.
^m^ Only identified as PIM if regularly used. Not identified as PIM in patients diagnosed with one of the following conditions: arthritis, leukaemia, lymphoma, breast cancer, prostate cancer, colorectal cancer, lung cancer, multiple sclerosis, eczema, psoriasis, Crohn’s disease, ulcerative colitis, and after organ transplantation.
^n^ Not counted as overuse in participants without osteoporosis.
^o^ B: in participants with estimated glomerular filtration rate (eGFR) < 30 ml/min/1.73m². C: in participants with eGFR ≥ 30 ml/min/1.73m². Only identified as PIM if eGFR was not missing prior to imputation.
^p^ Not identified as PIM in participants with one of the following conditions: dyslipidaemia, acute coronary syndromes, myocardial infarction, and stroke.
^q^ Not identified as PIM in participants with certain or possible diagnosis of epilepsy
^r^ Not identified as PIM in participants with self-reported depression or moderate to severe depression according to PHQ-9
^s^ Antiepileptics classified as FORTA B were not identified as PIM in participants with certain or possible diagnosis of epilepsy.
^t^ Only identified as PIM if used regularly.
^u^ Only identified as underuse in participants with TSH >8 mU/L.
^v^ Interview question: ‘How often do you get an influenza vaccination?’.

# Table S3. Comparison of baseline characteristics of study participants included in the subsample with complete vaccination information (N=5,575) and those excluded from it (N=48,721)

| **Characteristics** | **N (%)^a^** | |
| --- | --- | --- |
|  | **Vaccination subsample (N=5,575)** | **Study population not included in vaccination subsample (N=48,721)** |
| ***Sociodemographic/ -economic factors*** |  |  |
| Sex |  |  |
| Female | 2,884 (51.7) | 24,305 (49.9) |
| Male | 2,691 (48.3) | 24,416 (50.1) |
| Age group, years |  |  |
| 60 – 64 | 2,661 (47.7) | 23192 (47.6) |
| 65 – 69 | 2,432 (43.6) | 21689 (44.5) |
| ≥ 70 | 482 (8.7) | 3,840 (7.9) |
| Study centre |  |  |
| Augsburg | 688 (12.3) | 4,825 (9.9) |
| Regensburg | 120 (2.2) | 2,558 (5.3) |
| Mannheim | 729 (13.1) | 1,829 (3.8) |
| Freiburg | 373 (6.7) | 2,281 (4.7) |
| Saarbrücken | 324 (5.8) | 2,399 (4.9) |
| Essen | 249 (4.5) | 2,421 (5.0) |
| Münster | 23 (0.4) | 2,534 (5.2) |
| Düsseldorf | 10 (0.2) | 2,354 (4.8) |
| Halle | 229 (4.1) | 2,578 (5.3) |
| Leipzig | 886 (15.9) | 1,954 (4.0) |
| Berlin North | 244 (4.4) | 2,440 (5.0) |
| Central Berlin | 199 (3.6) | 2,573 (5.3) |
| Berlin South | 23 (0.4) | 2,695 (5.5) |
| Hannover | 769 (13.8) | 2,097 (4.3) |
| Hamburg | 99 (1.8) | 2,496 (5.1) |
| Bremen | 296 (5.3) | 2,457 (5.0) |
| Kiel | 50 (0.9) | 2,461 (5.1) |
| Neubrandenburg | 166 (3.0) | 3,235 (6.6) |
| Neustelitz | 98 (1.8) | 711 (1.5) |
| Waren (Müritz) | 0 (0.0) | 1,125 (2.3) |
| Demmin | 0 (0.0) | 698 (1.4) |
| Income^b^ |  |  |
| Low | 3,211 (57.6) | 27,268 (56.0) |
| Middle | 1,681 (30.2) | 15,185 (31.2) |
| High | 683 (12.3) | 6,268 (12.9) |
| Years of education |  |  |
| < 13 | 195 (3.5) | 2,056 (4.2) |
| ≥ 13 | 5,380 (96.5) | 46,665 (95.8) |
| Social network index^c^ |  |  |
| I+II | 2,881 (51.7) | 26,573 (54.5) |
| III+IV | 2,694 (48.3) | 22,148 (45.5) |
| ***Lifestyle factors*** |  |  |
| BMI *(kg/m²)* |  |  |
| < 20 | 104 (1.9) | 1073 (2.2) |
| 20 – 24.9 | 1,494 (26.8) | 13,075 (26.8) |
| 25 – 29.9 | 2,423 (43.5) | 20,305 (41.7) |
| ≥ 30 | 1,554 (27.9) | 14,268 (29.3) |
| Physical activity level^d^ |  |  |
| Insufficient | 725 (13.0) | 6,171 (12.7) |
| Sufficient | 4,850 (87.0) | 42,550 (87.3) |
| Alcohol consumption^e^ |  |  |
| Abstainer | 455 (8.2) | 4,671 (5.6) |
| Low | 4,460 (80.0) | 38,653 (79.3) |
| Moderate to high | 660 (11.8) | 5,397 (11.1) |
| Smoking status |  |  |
| Never smoker | 2,622 (47.0) | 20,992 (43.1) |
| Former smoker, years since quit |  |  |
| > 20 years | 1,342 (24.1) | 11,457 (23.5) |
| ≤ 20 years | 834 (15.0) | 7,976 (16.4) |
| Current smoker | 777 (13.9) | 8,296 (17.0) |
| ***Comorbidities*** |  |  |
| Diabetes | 635 (11.4) | 6,049 (12.4) |
| Arterial hypertension | 2,787 (50.0) | 24,174 (49.6) |
| Atrial fibrillation/arrhythmia | 830 (14.9) | 7,405 (15.2) |
| Stroke | 162 (2.9) | 1,717 (3.5) |
| Myocardial infarction | 158 (2.8) | 2,076 (4.3) |
| Heart failure | 298 (5.3) | 2,678 (5.5) |
| Acute coronary syndrome | 349 (6.3) | 4,042 (8.3) |
| Asthma | 395 (7.1) | 3,549 (7.3) |
| COPD | 428 (7.7) | 3,641 (7.5) |
| Osteoporosis | 409 (7.3) | 3,546 (7.3) |
| Thyroid disease/hypothyroidism^f^ | 1,667 (29.9) | 13,284 (27.3) |
| History of fractures^g^ | 983 (17.6) | 8,720 (17.9) |
| Pain^h^ | 3,679 (66.0) | 33,208 (68.2) |
| Parkinson’s disease | 14 (0.3) | 183 (0.4) |
| Cognitive impairment/dementia^i^ | 190 (3.4) | 2,044 (4.2) |
| History of depression^j^ | 835 (15.0) | 7,696 (15.8) |
| Epilepsy | 35 (0.6) | 361 (0.7) |
| Insomnia | 1,494 (26.8) | 12,342 (25.3) |
| Gastrointestinal symptoms^k^ | 1,718 (30.8) | 14,822 (30.4) |
| History of cancer | 814 (14.6) | 6,798 (14.0) |
| Renal impairment^l^ | 235 (4.2) | 2,493 (5.1) |

Values in bold are statistically significant (p<0.05).
Abbreviations: BMI, body mass index; COPD, chronic obstructive pulmonary disease; PIM, potentially inappropriate medication.

^a^ Calculated from first imputed dataset.
^b^ Average total household income before tax. Categories: low: <2900€; middle: 2900-<5000€; high: ≥5000€.
^c^ Categories: I (isolated) to IV (least isolated). ^d^ Adherence to WHO recommendations following Global Physical Activity Questionnaire (GPAQ). Categories: sufficient: ≥ 600 metabolic equivalent of task (MET)–min/week; insufficient: < 600 MET–min/week.
^e^ Alcohol consumption, g ethanol per day. Categories: abstainer: 0 g/day; low: women 0-19.99g/d or man 0-39.99g/d; moderate – high: women ≥ 20g/d or man ≥ 40g/d.
^f^ Lifetime history of thyroid disease, self-reported. Underuse for the indication hypothyroidism was only considered in individuals with TSH > 8.0 mU/L (cut-off for treatment of older patients suggested by FORTA list)
^g^ Fractures since aged 50 years.
^h^ Pain-related diagnoses included certain or likely migraine or tension headache in the last 12 months (following the 3rd edition of The International Classification of Headache Disorders), and self-reported information on arthritis, osteoarthritis, and gout.
^i^ Based on the interview question ‘Have you ever contacted a physician due to memory difficulties or are you planning to speak to a physician about it?’.
^j^ Lifetime history of depression, self-reported. ^k^ Combination of self-reported information on reflux, peptic ulcer (‘Have you ever been diagnosed with this condition by a physician?’), and regular intake of NSAIDs (ATC M01A). Underuse for this indication was only considered if the participant was diagnosed with reflux or peptic ulcer in the year of their baseline assessment and in participants regularly taking NSAIDs.
^l^ Estimated glomerular filtration rate <60ml/min/1.73m², calculated based on CKD-EPI equation.

# Table S4. Percentages of missing data of covariates prior to imputation in population analyzed (N=54,296)

| **Characteristics** | **Proportion of missing (%)** |
| --- | --- |
| ***Sociodemographic/ -economic factors*** |  |
| Sex | 0.0 |
| Age | 0.0 |
| Study centre | 0.0 |
| Income^a^ | 7.3 |
| Years of education | 0.2 |
| Social network index | 26.7 |
| ***Lifestyle factors*** |  |
| BMI *(kg/m^2^)* | 3.7 |
| Physical activity level *(MET-min/week)* | 8.8 |
| Alcohol consumption *(g/day)* | 7.6 |
| Smoking status | 5.3 |
| Quitage | 0.2 |
| ***Comorbidities*** | - |
| History of fractures^b^ | 26.8 |
| History of cancer | 0.4 |
| Renal impairment^c^ | 4.6 |

Abbreviations: BMI, body mass index; MET, metabolic equivalent of task.

^a^ Average total household income before tax. Categories: very low: <1700€; low: 1700-<2900€; middle: 2900-<5000€; high: ≥5000€.
^b^ Fractures since aged 50.
^c^ Estimated glomerular filtration rate < 60 ml/min/1.73m², calculated based on the CKD-EPI equation.

# Table S5. Percentages of missing data of indications for medication use prior to imputation in population analyzed (N=54,296)

| **FORTA Indication** | **Proportion of missing (%)** |
| --- | --- |
| Diabetes | 0.3 |
| Arterial hypertension | 0.4 |
| Atrial fibrillation/arrhythmia | 1.0 |
| Stroke | 0.4 |
| Myocardial infarction | 0.3 |
| Heart failure | 1.2 |
| Acute coronary syndrome | 0.8 |
| Asthma | 0.5 |
| COPD | 0.6 |
| Tiffeneau index^a^ | 29.1 |
| Osteoporosis | 0.9 |
| Thyroid disease/hypothyroidism^b^ | 0.5 |
| TSH^c^ *(mU/l)* | 48.0 |
| Pain^d^ | 4.6 |
| Parkinson’s disease | 0.2 |
| Cognitive impairment/dementia^e^ | 0.4 |
| History of depression | 0.6 |
| PHQ-9^f^ | 12.5 |
| Epilepsy | 0.3 |
| Insomnia | 24.7 |
| Gastrointestinal symptoms^g^ | 0.5 |

Abbreviations: COPD, chronic obstructive pulmonary disease; PHQ-9, Patient Health Questionnaire; TSH, thyroid stimulating hormone.

^a^ Proportion of missing values among participants with COPD: 20.5%. Participants with self-reported lifetime history of COPD but missing Tiffeneau Index were not classified as underusers for this specific indication.
^b^ Lifetime history of thyroid disease, self-reported. Underuse was only considered in individuals with TSH > 8.0 mU/L (cut-off for treatment of older patients suggested by FORTA list).
^c^ Proportion of missing values among participants with self-reported history of thyroid disease: 46.7%. Participants with a lifetime history of thyroid diseases but missing TSH blood levels were not classified as underusers for this specific indication. ^d^ Combination of self-reported pain in the last 4 weeks and pain-related diagnoses (including certain or likely migraine or tension headache in the last 12 months (following the 3rd edition of The International Classification of Headache Disorders), and self-reported information on arthritis, osteoarthritis, and gout).
^e^ Self-reported bad or very bad memory based on the interview question ‘How would you evaluate your memory at the moment?’ – five categories: very good to very bad.
^f^ PHQ-9 sum scores were used to determine the indication for depression treatment: PIM and overuse were considered in participants with self-reported lifetime history of depression or moderate to severe depression according to PHQ-9 (sum score ≥ 15). Underuse was only considered in untreated individuals with severe depression according to or PHQ-9 (sum score ≥ 20). Proportion of missing values among participants with a lifetime history of depression: 13.9%. Missing values were treated as PHQ-9 sum score < 15.
^g^ Combination of self-reported information on reflux, peptic ulcer (‘Have you ever been diagnosed with this condition by a physician?’), and regular intake of NSAIDs (ATC M01A)..

# Table S6. Potential factors associated with PIM, medication underuse, and overuse in the NAKO study - Results of the logistic regression model adjusted for age, sex, and study centre (N=54,296)

| **Variables** | **PIM** | | |  | **Underuse** | | |  | **Overuse** | | |
| --- | --- | --- | --- | --- | --- | --- | --- | --- | --- | --- | --- |
|  | **OR (95% CI)** | **p^a^** | **FDR^b^** |  | **OR (95% CI)** | **p^a^** | **FDR^b^** |  | **OR (95% CI)** | **p^a^** | **FDR^b^** |
| ***Sociodemographic/-economic factors***  Sex |  |  |  |  |  |  |  |  |  |  |  |
| Female | Ref |  |  |  | Ref |  |  |  | Ref |  |  |
| Male | **0.86 (0.83; 0.90)** | **<.001** | **<.001** |  | **0.79 (0.75; 0.82)** | **<.001** | **<.001** |  | **0.75 (0.72; 0.78)** | **<.001** | **<.001** |
| Age group, years |  |  |  |  |  |  |  |  |  |  |  |
| 60 – 64 | Ref |  |  |  | Ref |  |  |  | Ref |  |  |
| 65 – 69 | **1.22 (1.17; 1.27)** | **<.001** | **<.001** |  | 0.97 (0.93; 1.01) | 0.140 | 0.190 |  | **1.20 (1.15; 1.25)** | **<.001** | **<.001** |
| ≥ 70 | **1.43 (1.33; 1.54)** | **<.001** | **<.001** |  | 1.01 (0.93; 1.10) | 0.797 | 0.826 |  | **1.43 (1.33; 1.54)** | **<.001** | **<.001** |
| Study centre |  |  |  |  |  |  |  |  |  |  |  |
| Augsburg | Ref |  |  |  | Ref |  |  |  | Ref |  |  |
| Regensburg | **1.15 (1.04; 1.28)** | **0.008** | **0.010** |  | **0.87 (0.77; 0.98)** | **0.017** | **0.031** |  | **1.13 (1.02; 1.26)** | **0.023** | **0.035** |
| Mannheim | 0.96 (0.86; 1.07) | 0.456 | 0.473 |  | 0.88 (0.78; 0.99) | 0.040 | 0.061 |  | **0.88 (0.79; 0.99)** | **0.029** | **0.042** |
| Freiburg | **0.74 (0.66; 0.82)** | **<.001** | **<.001** |  | 1.08 (0.96; 1.21) | 0.191 | 0.241 |  | **1.18 (1.07; 1.32)** | **0.002** | **0.004** |
| Saarbrücken | 1.00 (0.90; 1.11) | 0.971 | 0.988 |  | 0.98 (0.87; 1.10) | 0.696 | 0.735 |  | **1.14 (1.02; 1.26)** | **0.021** | **0.033** |
| Essen | 1.11 (1.00; 1.24) | 0.043 | 0.051 |  | 1.07 (0.95; 1.19) | 0.265 | 0.322 |  | 1.06 (0.95; 1.18) | 0.336 | 0.420 |
| Münster | **0.80 (0.71; 0.89)** | **<.001** | **<.001** |  | 0.92 (0.82; 1.03) | 0.159 | 0.211 |  | **1.23 (1.10; 1.37)** | **<.001** | **0.001** |
| Düsseldorf | 0.94 (0.84; 1.05) | 0.284 | 0.311 |  | 0.92 (0.81; 1.04) | 0.173 | 0.224 |  | 1.05 (0.95; 1.17) | 0.330 | 0.420 |
| Halle | **1.52 (1.38; 1.68)** | **<.001** | **<.001** |  | **0.81 (0.72; 0.91)** | **<.001** | **0.001** |  | **0.86 (0.77; 0.96)** | **0.008** | **0.014** |
| Leipzig | **1.41 (1.28; 1.56)** | **<.001** | **<.001** |  | 0.96 (0.86; 1.08) | 0.501 | 0.549 |  | **0.85 (0.76; 0.95)** | **0.005** | **0.009** |
| Berlin North | 0.96 (0.86; 1.06) | 0.408 | 0.431 |  | 0.88 (0.78; 0.99) | 0.034 | 0.056 |  | 0.94 (0.85; 1.05) | 0.290 | 0.393 |
| Central Berlin | 0.90 (0.81; 1.00) | 0.049 | 0.057 |  | 0.94 (0.84; 1.06) | 0.318 | 0.370 |  | 0.96 (0.86; 1.07) | 0.446 | 0.541 |
| Berlin South | **0.79 (0.71; 0.88)** | **<.001** | **<.001** |  | 0.89 (0.79; 1.00) | 0.051 | 0.074 |  | 0.95 (0.85; 1.06) | 0.339 | 0.420 |
| Hannover | 0.91 (0.82; 1.01) | 0.088 | 0.100 |  | **0.87 (0.78; 0.98)** | **0.019** | **0.032** |  | 0.97 (0.87; 1.08) | 0.574 | 0.654 |
| Hamburg | **0.74 (0.66; 0.83)** | **<.001** | **<.001** |  | 0.90 (0.80; 1.01) | 0.081 | 0.116 |  | **0.77 (0.68; 0.86)** | **<.001** | **<.001** |
| Bremen | **0.81 (0.73; 0.91)** | **<.001** | **<.001** |  | **0.88 (0.78; 0.98)** | **0.025** | **0.042** |  | 1.02 (0.92; 1.14) | 0.690 | 0.764 |
| Kiel | 1.00 (0.90; 1.11) | 0.990 | 0.990 |  | **0.83 (0.74; 0.94)** | **0.004** | **0.006** |  | 1.00 (0.9; 1.10) | 0.924 | 0.975 |
| Neubrandenburg | **1.47 (1.34; 1.62)** | **<.001** | **<.001** |  | **0.76 (0.68; 0.85)** | **<.001** | **<.001** |  | 0.90 (0.75; 1.08) | 0.246 | 0.341 |
| Neustelitz | **1.39 (1.19; 1.63)** | **<.001** | **<.001** |  | 0.90 (0.74; 1.08) | 0.249 | 0.296 |  | 1.00 (0.86; 1.16) | 0.975 | 0.992 |
| Waren (Müritz) | **1.44 (1.25; 1.66)** | **<.001** | **<.001** |  | **0.57 (0.48; 0.69)** | **<.001** | **<.001** |  | 1.10 (0.92; 1.32) | 0.304 | 0.402 |
| Demmin | **1.71 (1.45; 2.02)** | **<.001** | **<.001** |  | 0.80 (0.65; 0.99) | 0.036 | 0.057 |  | **1.13 (1.02; 1.26)** | **0.023** | **0.035** |
| Income^c^ |  |  |  |  |  |  |  |  |  |  |  |
| Low | Ref |  |  |  | Ref |  |  |  | Ref |  |  |
| Middle | **0.70 (0.67; 0.73)** | **<.001** | **<.001** |  | **0.84 (0.80; 0.89)** | **<.001** | **<.001** |  | **0.90 (0.86; 0.95)** | **<.001** | **<.001** |
| High | **0.55 (0.51; 0.59)** | **<.001** | **<.001** |  | **0.79 (0.73; 0.85)** | **<.001** | **<.001** |  | **0.92 (0.86; 0.98)** | **0.023** | **0.035** |
| Years of education |  |  |  |  |  |  |  |  |  |  |  |
| < 13 | Ref |  |  |  | Ref |  |  |  | Ref |  |  |
| ≥ 13 | **0.63 (0.57; 0.69)** | **<.001** | **<.001** |  | **0.63 (0.57; 0.69)** | **<.001** | **<.001** |  | **0.63 (0.57; 0.69)** | **<.001** | **<.001** |
| Social network index^d^ |  |  |  |  |  |  |  |  |  |  |  |
| I+II | Ref |  |  |  | Ref |  |  |  | Ref |  |  |
| III+IV | **0.83 (0.79; 0.86)** | **<.001** | **<.001** |  | **0.89 (0.84; 0.94)** | **<.001** | **<.001** |  | **0.91 (0.86; 0.97)** | **0.003** | **0.007** |
| ***Lifestyle factors*** |  |  |  |  |  |  |  |  |  |  |  |
| BMI, kg/m² |  |  |  |  |  |  |  |  |  |  |  |
| < 20 | 0.92 (0.78; 1.08) | 0.292 | 0.314 |  | 1.09 (0.94; 1.27) | 0.236 | 0.293 |  | 1.03 (0.89; 1.19) | 0.697 | 0.764 |
| 20 – 24.9 | Ref |  |  |  | Ref |  |  |  | Ref |  |  |
| 25 – 29.9 | **1.50 (1.43; 1.59)** | **<.001** | **<.001** |  | 1.00 (0.95; 1.06) | 0.898 | 0.898 |  | **1.07 (1.02; 1.13)** | **0.006** | **0.012** |
| ≥ 30 | **2.77 (2.62; 2.92)** | **<.001** | **<.001** |  | 1.02 (0.96; 1.08) | 0.576 | 0.619 |  | **1.32 (1.25; 1.39)** | **<.001** | **<.001** |
| Physical activity level^e^ |  |  |  |  |  |  |  |  |  |  |  |
| Insufficient | Ref |  |  |  | Ref |  |  |  | Ref |  |  |
| Sufficient | **0.71 (0.67; 0.75)** | **<.001** | **<.001** |  | **0.71 (0.67; 0.75)** | **<.001** | **<.001** |  | **0.71 (0.67; 0.75)** | **<.001** | **<.001** |
| Alcohol consumption^f^ |  |  |  |  |  |  |  |  |  |  |  |
| Abstainer | **1.67 (1.57; 1.79)** | **<.001** | **<.001** |  | **1.21 (1.12; 1.30)** | **<.001** | **<.001** |  | **1.42 (1.33; 1.52)** | **<.001** | **<.001** |
| Low | Ref |  |  |  | Ref |  |  |  | Ref |  |  |
| Moderate to high | 1.05 (0.99; 1.12) | 0.126 | 0.142 |  | 1.03 (0.97; 1.11) | 0.340 | 0.388 |  | **1.09 (1.02; 1.17)** | **0.008** | **0.014** |
| Smoking |  |  |  |  |  |  |  |  |  |  |  |
| Never | Ref |  |  |  | Ref |  |  |  | Ref |  |  |
| Former smoker, years since quit |  |  |  |  |  |  |  |  |  |  |  |
| > 20 years | **1.09 (1.03; 1.14)** | **0.001** | **0.001** |  | 1.06 (1.00; 1.12) | 0.048 | 0.072 |  | **1.07 (1.02; 1.13)** | **0.011** | **0.018** |
| ≤ 20 years | **1.28 (1.20; 1.35)** | **<.001** | **<.001** |  | **1.16 (1.09; 1.24)** | **<.001** | **<.001** |  | **1.40 (1.32; 1.48)** | **<.001** | **<.001** |
| Current smoker | **1.26 (1.19; 1.34)** | **<.001** | **<.001** |  | **1.12 (1.05; 1.29)** | **<.001** | **<.001** |  | **1.31 (1.23; 1.39)** | **<.001** | **<.001** |
| ***Comorbidities*** |  |  |  |  |  |  |  |  |  |  |  |
| Diabetes | **2.93 (2.78; 3.09)** | **<.001** | **<.001** |  | **1.19 (1.12; 1.27)** | **<.001** | **<.001** |  | **1.55 (1.46; 1.64)** | **<.001** | **<.001** |
| Arterial hypertension | **5.21 (4.98; 5.44)** | **<.001** | **<.001** |  | **1.40 (1.34; 1.46)** | **<.001** | **<.001** |  | **1.20 (1.15; 1.25)** | **<.001** | **<.001** |
| Atrial fibrillation/arrhythmia | **1.98 (1.89; 2.08)** | **<.001** | **<.001** |  | **5.54 (5.26; 5.83)** | **<.001** | **<.001** |  | **0.93 (0.88; 0.98)** | **0.012** | **0.020** |
| Stroke | **2.40 (2.18; 2.63)** | **<.001** | **<.001** |  | **1.62 (1.46; 1.80)** | **<.001** | **<.001** |  | **1.43 (1.29; 1.58)** | **<.001** | **<.001** |
| Myocardial infarction | **1.72 (1.57; 1.88)** | **<.001** | **<.001** |  | 0.99 (0.88; 1.11) | 0.835 | 0.850 |  | **1.22 (1.11; 1.35)** | **<.001** | **<.001** |
| Heart failure | **2.17 (2.01; 2.34)** | **<.001** | **<.001** |  | **2.10 (1.93; 2.28)** | **<.001** | **<.001** |  | **1.45 (1.34; 1.57)** | **<.001** | **<.001** |
| Acute coronary syndromes | **1.55 (1.45; 1.65)** | **<.001** | **<.001** |  | **1.18 (1.09; 1.28)** | **<.001** | **<.001** |  | **1.13 (1.05; 1.22)** | **0.001** | **0.003** |
| Asthma | **1.39 (1.29; 1.49)** | **<.001** | **<.001** |  | **1.65 (1.53; 1.77)** | **<.001** | **<.001** |  | 1.08 (1.00; 1.16) | 0.057 | 0.081 |
| COPD | **1.90 (1.78; 2.03)** | **<.001** | **<.001** |  | **2.46 (2.29; 2.63)** | **<.001** | **<.001** |  | **1.24 (1.15; 1.33)** | **<.001** | **<.001** |
| Osteoporosis | **1.60 (1.49; 1.71)** | **<.001** | **<.001** |  | **8.61 (8.02; 9.23)** | **<.001** | **<.001** |  | 0.98 (0.90; 1.05) | 0.508 | 0.591 |
| Thyroid disease/hypothyroidism^g^ | **1.31 (1.25; 1.37)** | **<.001** | **<.001** |  | **1.17 (1.12; 1.23)** | **<.001** | **<.001** |  | **1.15 (1.10; 1.21)** | **<.001** | **<.001** |
| History of fractures^h^ | **1.10 (1.03; 1.16)** | **0.003** | **0.004** |  | **1.46 (1.37; 1.55)** | **<.001** | **<.001** |  | 0.98 (0.92; 1.04) | 0.468 | 0.555 |
| Pain^i^ | **1.85 (1.76; 1.93)** | **<.001** | **<.001** |  | **1.76 (1.67; 1.85)** | **<.001** | **<.001** |  | 1.00 (0.96; 1.04) | 0.999 | 0.999 |
| Parkinson’s disease | **11.72 (8.24; 16.68)** | **<.001** | **<.001** |  | **2.74 (2.06; 3.66)** | **<.001** | **<.001** |  | **1.46 (1.08; 1.98)** | **0.015** | **0.024** |
| Dementia^j^ | **2.10 (1.93; 2.30)** | **<.001** | **<.001** |  | **1.87 (1.70; 2.05)** | **<.001** | **<.001** |  | 1.02 (0.92; 1.12) | 0.748 | 0.805 |
| Depression^k^ | **2.56 (2.44; 2.69)** | **<.001** | **<.001** |  | **1.58 (1.49; 1.67)** | **<.001** | **<.001** |  | **1.69 (1.60; 1.77)** | **<.001** | **<.001** |
| Epilepsy | **4.45 (3.63; 5.46)** | **<.001** | **<.001** |  | 1.21 (0.95; 1.54) | 0.118 | 0.164 |  | **1.75 (1.42; 2.15)** | **<.001** | **<.001** |
| Insomnia | **1.26 (1.20; 1.31)** | **<.001** | **<.001** |  | **1.28 (1.22; 1.34)** | **<.001** | **<.001** |  | 1.00 (0.95; 1.05) | 0.962 | 0.992 |
| Gastrointestinal symptoms^l^ | **1.78 (1.71; 1.86)** | **<.001** | **<.001** |  | **1.89 (1.80; 1.97)** | **<.001** | **<.001** |  | **1.07 (1.02; 1.12)** | **0.002** | **0.005** |
| History of cancer | **1.18 (1.11; 1.24)** | **<.001** | **<.001** |  | **1.12 (1.06; 1.19)** | **<.001** | **<.001** |  | **1.16 (1.10; 1.22)** | **<.001** | **<.001** |
| Renal impairment^m^ | **2.33 (2.15; 2.54)** | **<.001** | **<.001** |  | 0.95 (0.86; 1.05) | 0.353 | 0.395 |  | **1.61 (1.47; 1.75)** | **<.001** | **<.001** |

Values in bold are statistically significant with FDR (p<0.05).
Abbreviations: BMI, body mass index; CI, confidence interval; COPD, chronic obstructive pulmonary disease; FDR, false discovery rate; OR, odds ratio; PIM, potentially inappropriate medication; Ref, reference.

^a^ Unadjusted for multiple testing.
^b^ P values adjusted for multiple testing using the false discovery rate (Benjamini-Hochberg method).
^c^ Average total household income before tax. Categories: low: <2900€; middle: 2900-<5000€; high: ≥5000€.
^d^ Categories: I (isolated) to IV (least isolated).
^e^ Adherence to WHO recommendations following Global Physical Activity Questionnaire (GPAQ). Categories: sufficient: ≥ 600 metabolic equivalent of task (MET) –min/week; insufficient: < 600 MET–min/week. ^f^ Alcohol consumption, g ethanol per day. Categories: abstainer: 0 g/day; low: women 0-19.99g/d or man 0-39.99g/d; moderate – high: women ≥ 20g/d or man ≥ 40g/d.
^g^ Lifetime history of thyroid disease, self-reported. Underuse was only considered in individuals with TSH > 8.0 mU/L (cut-off for treatment of older patients suggested by FORTA list).
^h^ Fractures since aged 50.
^i^ Pain-related diagnoses included certain or likely migraine or tension headache in the last 12 months (following the 3rd edition of The International Classification of Headache Disorders), and self-reported information on arthritis, osteoarthritis, and gout.
^j^ Based on the interview question ‘Have you ever contacted a physician due to memory difficulties or are you planning to speak to a physician about it?’.
^k^ Lifetime history of depression, self-reported.
^l^ Combination of self-reported information on reflux, peptic ulcer (‘Have you ever been diagnosed with this condition by a physician?’), and regular intake of NSAIDs (ATC M01A). Underuse was only considered if the participant was diagnosed with reflux or peptic ulcer in the year of their baseline assessment and in participants regularly taking NSAIDs. ^m^ Estimated glomerular filtration rate <60 ml/min/1.73m², calculated based on CKD-EPI equation.
Note: PIM exposure was defined according to the “Fit fOR The Aged” (FORTA) list 2024.

# References

1. van Buuren S. Multiple imputation of discrete and continuous data by fully conditional specification. *Stat Methods Med Res* 2007, **16**(3):219-242. doi: 10.1177/0962280206074463

2. van Buuren S. *Flexible Imputation of Missing Data*, 2nd edn. New York: Chapman & Hall/CRC; 2018.

3. Abayomi K, Gelman A, Levy M. Diagnostics for Multivariate Imputations. *Journal of the Royal Statistical Society Series C: Applied Statistics* 2008, **57**(3):273-291. doi: 10.1111/j.1467-9876.2007.00613.x
